# Exploring the Antitumor Potential of Copper Complexes Based on Ester Derivatives of Bis(pyrazol-1-yl)acetate Ligands

**DOI:** 10.3390/ijms23169397

**Published:** 2022-08-20

**Authors:** Maura Pellei, Carlo Santini, Luca Bagnarelli, Chiara Battocchio, Giovanna Iucci, Iole Venditti, Carlo Meneghini, Simone Amatori, Paolo Sgarbossa, Cristina Marzano, Michele De Franco, Valentina Gandin

**Affiliations:** 1 School of Science and Technology, Chemistry Division, University of Camerino, Via S. Agostino 1, 62032 Camerino, Italy; 2 Department of Science, Roma Tre University, Via della Vasca Navale 79, 00146 Roma, Italy; 3 Department of Industrial Engineering, University of Padova, Via Marzolo 9, 35131 Padova, Italy; 4 Department of Pharmaceutical and Pharmacological Sciences, University of Padova, Via Marzolo 5, 35131 Padova, Italy

**Keywords:** copper, bis(pyrazolyl)acetate ligands, cytotoxicity, SR-XPS, XAS

## Abstract

Bis(pyrazol-1-yl)acetic acid (HC(pz)_2_COOH) and bis(3,5-dimethyl-pyrazol-1-yl)acetic acid (HC(pz^Me2^)_2_COOH) were converted into the methyl ester derivatives **1** (L^OMe^) and **2** (L^2OMe^), respectively, and were used for the preparation of Cu(I) and Cu(II) complexes **3**–**10**. The copper(II) complexes were prepared by the reaction of CuCl_2_·2H_2_O or CuBr_2_ with ligands **1** and **2** in methanol solution. The copper(I) complexes were prepared by the reaction of Cu[(CH_3_CN)_4_]PF_6_ and 1,3,5-triaza-7-phosphaadamantane (PTA) or triphenylphosphine with L^OMe^ and L^2OMe^ in acetonitrile solution. Synchrotron radiation-based complementary techniques (XPS, NEXAFS, and XAS) were used to investigate the electronic and molecular structures of the complexes and the local structure around copper ions in selected Cu(I) and Cu(II) coordination compounds. All Cu(I) and Cu(II) complexes showed a significant in vitro antitumor activity, proving to be more effective than the reference drug cisplatin in a panel of human cancer cell lines, and were able to overcome cisplatin resistance. Noticeably, Cu complexes appeared much more effective than cisplatin in 3D spheroid cultures. Mechanistic studies revealed that the antitumor potential did not correlate with cellular accumulation but was consistent with intracellular targeting of PDI, ER stress, and paraptotic cell death induction.

## 1. Introduction

Copper is an essential element in all living organisms and is a crucial trace element in redox chemistry, growth, and development [1]. Metallic copper and its coordination complexes were found to be highly active against bacteria, yeasts, fungi, and viruses [2,3]. Being involved in a wide spectrum of biochemical processes also due to its flexible redox behavior, copper accumulates in tumors [4,5,6,7,8,9] owing to the selective permeability of the cancer cell membranes [10]. Cu(I) and Cu(II) complexes have recently received great attention for obtaining more potent, clinically effective, and less toxic metal-based antiproliferative drugs with unique antitumor properties [7,11,12,13,14,15]. Even if few studies have reported on their exact molecular mechanism of action, several proteins or key enzymes have emerged as suitable targets [16]. This ability to target biomolecules other than DNA confers them a broader spectrum of activity and a better toxicity profile, thereby providing the possibility of circumventing the problems faced with platinum-based drugs, such as dose-limiting toxicity and inherent or acquired resistance [17,18,19,20].

We have recently reported that copper complexes with heteroscorpionate ligands, obtained by conjugation of bis(pyrazolyl)acetates with nitroimidazole, glucosamine, and a non-competitive NMDA receptor antagonist, showed cytotoxic activity towards a panel of several human tumor cell lines [21,22,23,24]. Bis(pyrazol-1-yl)- and bis(3,5-dimethylpyrazol-1-yl)-acetates were also conjugated with the 2-hydroxyethylester and 2-aminoethylamide derivatives of the antineoplastic drug lonidamine to prepare Cu(I) and Cu(II) complexes that might act through synergistic mechanisms of action due to the presence of lonidamine and copper in the same chemical entity [25].

Therefore, the aim of this work was to study the capability of smaller ester derivatives of bis(pyrazolyl)acetates species to form Cu(I) and Cu(II) complexes potentially useful for anticancer purposes.

**Figure 1:**
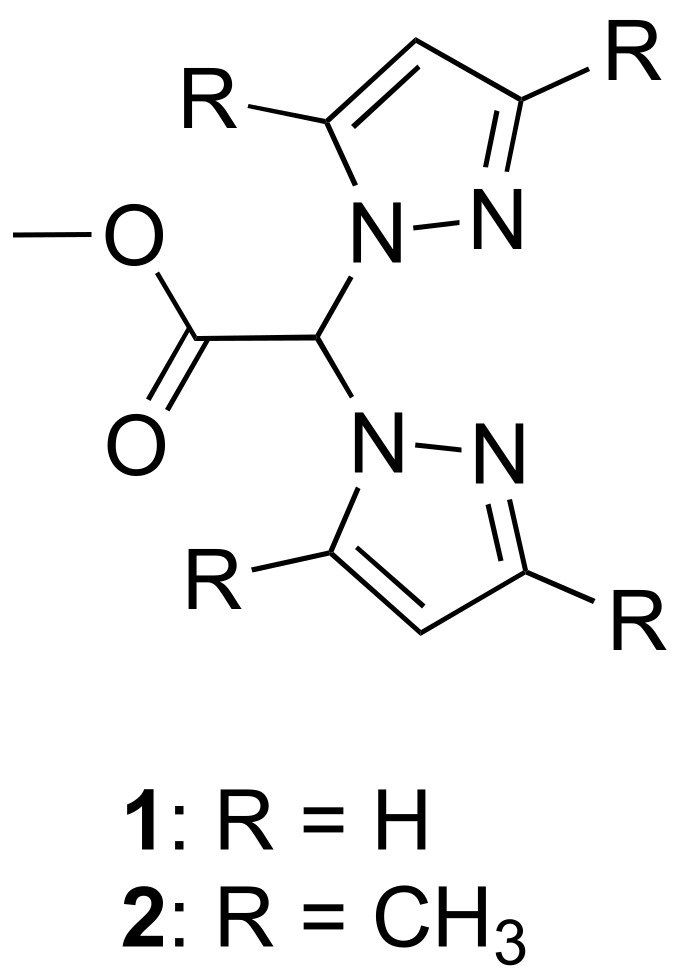
Chemical structures of ligands **1** and **2**.

**Figure 2:**
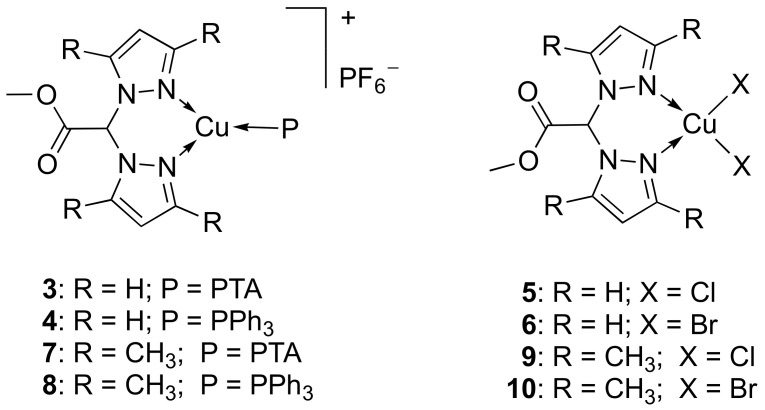
Chemical structures of complexes **3**–**10**.

Bis(pyrazol-1-yl)acetic acid (HC(pz)_2_COOH) and bis(3,5-dimethyl-pyrazol-1-yl)acetic acid (HC(pz^Me2^)_2_COOH) were converted into the methyl ester derivatives **1** (L^OMe^) and **2** (L^2OMe^), respectively (Figure 1). Ligands **1** and **2** were used for the preparation of the Cu(I) and Cu(II) complexes **3**–**10** (Figure 2). Concerning the Cu(I) complexes, the lipophilic triphenylphosphine (PPh_3_) and the hydrophilic 1,3,5-triaza-7-phosphaadamantane (PTA), able to stabilize copper in +1 oxidation state, were selected as coligands in order to confer different solubility features to the corresponding metal complexes.

The molecular and electronic structure of selected Cu(I) and Cu(II) coordination compounds were examined in the solid state by means of synchrotron radiation-induced X-ray photoelectron spectroscopy (SR-XPS), near edge X-ray absorption fine structure (NEXAFS), and X-ray absorption (XAS) spectroscopies. This approach encompassing several different and complementary techniques allowed us to probe the copper ion oxidation state stability, to properly describe its coordination geometry, and to establish the molecular structure stability of the ligands upon interaction with the metal center [25,26,27].

The newly developed Cu(I) and Cu(II) complexes **3**–**10** and the corresponding uncoordinated ligands (**1** and **2**) were evaluated for their cytotoxic potential on a panel of different human cancer cell lines by means of both 2D and 3D cell viability studies. Mechanistic investigation allowed for deeply analyzing the main molecular and cellular determinants accounting for their anticancer activity. Additionally, microscopic analysis were performed in order to assess their ability to induce cancer cell death by means of an ER-stress driven, apoptosis-alternative cancer cell death, termed as paraptosis.

## 2. Results and Discussion

### 2.1. Synthesis and Characterization

The ligands [HC(pz)_2_COOCH_3_] (L^OMe^, **1**) and [HC(pz^Me2^)_2_COOCH_3_] (L^2OMe^, **2**) were prepared by a method described in the literature and were fully characterized. Chemical structures of the ligands are characterized by different residues R being H or CH_3_ in ligand **1** and **2**, respectively (Figure 1).

The Cu(I) complexes [(PTA)Cu(L^OMe^)]PF_6_ (**3**) and [(PTA)Cu(L^2OMe^)]PF_6_ (**7**) were prepared from the reaction of PTA, the Cu(I) salt Cu[(CH_3_CN)_4_]PF_6_, and the ligands L^OMe^ and L^2OMe^, respectively, following a one-pot synthesis in CH_3_CN as solvent. Analogously, the Cu(I) complexes [(PPh_3_)Cu(L^OMe^)]PF_6_ (**4**) and [(PPh_3_)Cu(L^2OMe^)]PF_6_ (**8**) were prepared from the reaction of PPh_3_, Cu(CH_3_CN)_4_PF_6_, and the related ligands (Figure 2).

All the compounds were soluble in CH_3_CN and DMSO; the complexes with PPh_3_ as a coligand (**4** and **8**) were also soluble in methanol, acetone, and CHCl_3_, while the complexes with PTA as a coligand (**3** and **7**) were soluble in methanol and acetone; only compound **3** was soluble in water. The IR spectra carried out on solid samples of the Cu(I) complexes showed all the expected bands for the chelating ligand and the phosphane coligand. The absorptions due to the C=O stretching of the ester groups were in the range 1759–1768 cm^−1^ and they did not significantly vary with respect to the free ligands. In a lower frequency region, complexes showed a broad strong band at 830–836 cm^−1^ due to the stretching vibrations of the PF_6_^−^ anion. The δ(PF_6_) bending vibrations in the spectra of all hexafluorophosphate complexes were observed as a narrow strong band at 555–558 cm^−1^. The ^1^H-NMR spectra of the Cu(I) complexes, recorded in CD_3_CN solution at room temperature, showed a single set of resonances for the pyrazole rings, indicating that the pyrazole protons were equivalent, with a slight shift due to the coordination to the metal center. The PTA and PPh_3_ coligands showed a characteristic series of peaks at δ 4.09–4.58 and 7.34–7.53 ppm, respectively, with an integration which confirmed the 1:1 stoichiometric ratio between the ligand and phosphane coligand. The room temperature ^31^P{H}-NMR spectra of the Cu(I) complexes, recorded in CD_3_CN solution, gave singlets shifted downfield with respect to the value of the free phosphanes PTA and PPh_3_ (δ = −102.07 and −4.85 ppm, respectively). In particular, the spectra of [(PTA)Cu(L^OMe^)]PF_6_ (**3**) and [(PTA)Cu(L^2OMe^)]PF_6_ (**7**) gave broad singlets centered at δ −93.52 and −95.72 ppm, respectively, while the spectra of [(PPh_3_)Cu(L^OMe^)]PF_6_ (**4**) and [(PPh_3_)Cu(L^2OMe^)]PF_6_ (**8**) exhibited broad singlets centered at δ −1.48 and −2.08 ppm, respectively. In all the spectra, the characteristic septets centered between −143.51 and −144.78 ppm were due to the PF_6_^−^ counterion.

The ESI-MS studies, performed by dissolving the Cu(I) complexes in CH_3_CN and recording the spectra in positive- and negative-ion mode, confirmed the formation of the PTA and PPh_3_ complexes and the presence of hexafluorophosphate as counterion. In particular, the formation of complexes **3**, **4**, **7**, and **8** was confirmed by the presence in the positive-ion ESI-MS spectra of the major peaks attributable to the [(PTA)Cu(L^OMe^)]^+^, [(PPh_3_)Cu(L^OMe^)]^+^, [(PTA)Cu(L^2OMe^)]^+^, and [(PPh_3_)Cu(L^2OMe^)]^+^ species, respectively. In the negative-ion spectra, [PF_6_]^−^ was observed as the major peak for all the complexes. The elemental analyses confirmed the stoichiometry and the purity of the products.

The copper(II) complexes [(L^OMe^)CuCl_2_] (**5**) and [(L^2OMe^)CuCl_2_] (**9**) were prepared by reaction of CuCl_2_·2H_2_O with [HC(pz)_2_COOCH_3_] (L^OMe^, **1**) and [HC(pz^Me2^)_2_COOCH_3_] (L^2OMe^, **2**), respectively, in methanol solution at room temperature (Figure 1). The copper(II) complexes [(L^OMe^)CuBr_2_] (**6**) and [(L^2OMe^)CuBr_2_] (**10**) were prepared from the reaction of CuBr_2_ with L^OMe^ (**1**) and L^2OMe^ (**2**), respectively, in methanol solution at room temperature (Figure 2). The syntheses were also performed starting from a 1:2 stoichiometric ratio between metal and ligand, obtaining the same products. The Cu(II) compounds **5**, **6**, **9**, and **10** were soluble in methanol, DMSO, and water and were air-stable even as solutions. The authenticity of compounds **5**, **6**, **9**, and **10** was confirmed by elemental analysis, IR spectroscopy, and electrospray mass spectra. The infrared spectra showed all the bands required by the presence of the chelating donor. Medium or strong absorptions at 1748–1761 cm^−1^, due to the carbonylic asymmetric stretching, were in the same range observed for the free ligands (1753 and 1760 cm^−1^ for L^OMe^ and L^2OMe^, respectively). In the spectra of compounds **5**, **6**, **9**, and **10**, weak absorptions due to the CH stretching were observed at 2922–3150 cm^−1^, while medium bands in the range 1510–1562 cm^−1^ were attributable to the C=C/C=N stretching vibrations. We observed the copper chloride stretching frequencies of complexes **5** and **9** as very strong absorptions at 278–279 cm^−1^. Analogously, the copper bromide stretching frequencies of complexes **6** and **10** appeared as strong absorptions at 229–235 cm^−1^. The ESI-MS study was conducted by dissolving the Cu(II) complexes compounds in methanol and recording the spectra in positive- and negative-ion mode. In the positive-ion spectrum of **5**, it was possible to detect the peaks at *m*/*z* 269 and 475 attributable to the [(L^OMe^ − H)Cu]^+^ and [(L^OMe^)Cu(L^OMe^ − H)]^+^ species, confirming the complex formation, while in the spectrum of **6**, the peaks at *m*/*z* 269 and 350 were consistent with the [(L^OMe^ − H)Cu]^+^ and [(L^OMe^)CuBr]^+^ species. In the positive-ion spectrum of **9**, a peak at *m*/*z* 420 was attributable to the [(L^2OMe^)CuCl_2_ + Na]^+^ adduct, confirming the complex formation, while in the spectrum of **10**, peaks at *m*/*z* 572 and 587 corresponded to the [Cu(L^2OMe^)(L^2OMe^ − Me)]^+^ and [Cu(L^2OMe^)(L^2OMe^ − H)]^+^ aggregates. The negative-ion mode spectra of the copper(II) chlorides and bromides showed peaks at *m*/*z* 170 and 304, attributable to the species [CuCl_3_]^−^ and [CuBr_3_]^−^, respectively, confirming the presence of the halides in the complexes.

### 2.2. Molecular and Electronic Structure in Solid State: XPS

With the aim to investigate the electronic and molecular structure of the copper coordination compounds in the solid state, XPS measurements were carried out on [HC(pz^Me2^)_2_COOCH_3_] (L^2OMe^, **2**), representative for both ligands **1** and **2** (the only structural difference being R = H in **1** and R = CH_3_ in **2**), on Cu(I) complex [(PPh_3_)Cu(L^2OMe^)]PF_6_ (**8**) and on Cu(II) complexes [(L^OMe^)CuCl_2_] (**5**) and [(L^2OMe^)CuCl_2_] (**9**).

XPS measurements were carried out at C1s, N1s, O1s, and Cu2p, and when necessary, Cl2p and P2p core levels; a complete collection of binding energy (BE), full width half maxima (FWHM), atomic ratios values, and assignments confirming the molecular structures as proposed for the coordination compounds and the stability of the ligand molecular structure upon coordination to copper is reported in Table S1 in the Supporting Information; here, only the most significative signals are discussed in detail.

**Figure 3:**
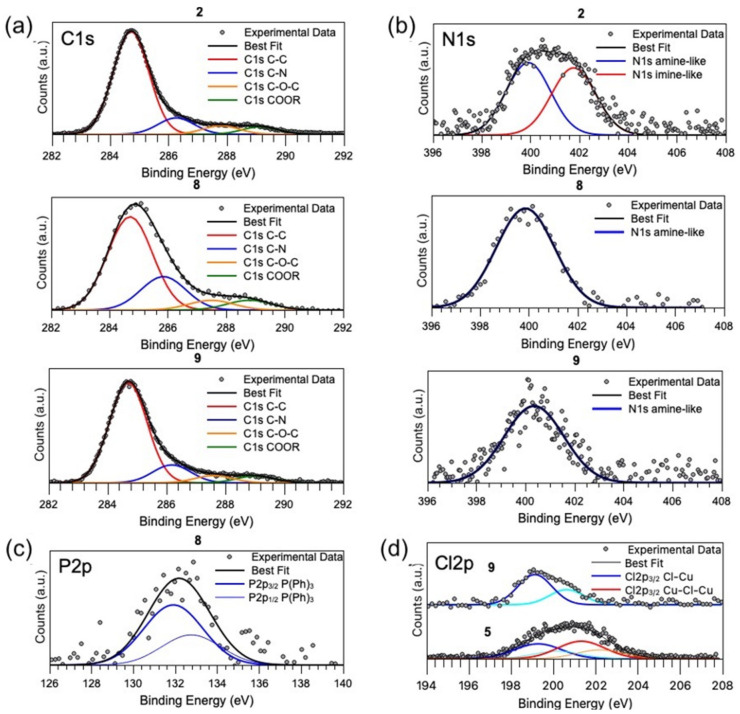
(**a**) C1s XPS spectra of **2** (top), **8** (middle), and **9** (bottom); (**b**) N1s XPS spectra of **2** (top), **8** (middle), and **9** (bottom); (**c**) P2p spectrum of complex **8**; (**d**) Cl2p spectra of **9** (top) and **5** (bottom).

The C1s signals collected on the four samples appeared composite, and by applying a curve-fitting procedure, it was possible to individuate at least four components assigned to different carbon atoms, as expected from the molecular structures: aromatic (plus a small contribution arising by aliphatic) C-C at 284.7 eV BE, C-N of the pyrazole ring at about 286 eV BE, C-O-C around 287 eV BE, and COOC at 289 eV. The C1s spectra, as expected, had similar composition for the ligand and the three coordination compounds, confirming the molecular stability of the ligand upon coordination with either Cu(I) or Cu(II) ions. C1s spectra of **2**, **8**, and **9** are reported in Figure 3a. For all samples, the atomic ratio C-O-C/COOC was 1/1, coherently with the proposed molecular structure; in addition, the relative amount of C-C observed in complex **9** was higher than the same contribution reported for complex **5**, as expected (R = H in **5**, R = CH_3_ in **9**) (see Table S1).

The N1s spectra reported in Figure 3b for ligand **2** and complexes **8** and **9** confirmed the chemical stability of the ligand and the coordination compound formation. Two spectral components could be individuated in the N1s spectrum of ligand **2**, as expected for pyrazole rings: the component at lower BE (399.9 eV) was attributed to amine-like nitrogen, and the peak at higher BE (401.8 eV) to imine-like N atoms [25,26,27,28]. On the other hand, the N1s spectra of complexes **8**, **5**, and **9** showed a single component at about 400 eV, corresponding to the symmetrized amine-like contributions, as expected for heterocycles coordinating metal ions [25,26,27,29,30].

The Cu2p spectra collected on the three complexes confirmed the presence of respectively Cu(I) (complex **8**, Cu2p_3/2_ BE = 932.2 eV) [31,32] and Cu(II) (complexes **5** and **9**: Cu2p_3/2_ BE = 935 eV) [33] ions. Cu2p spectra are reported in Figure S1 in the Supporting Information. In addition, P2p spectrum collected on complex **8** (Figure 3c) confirmed the presence of a stable PPh_3_ ligand coordinating the Cu(I) ion (P2p_3/2_ BE = 131.9 eV, coherently with literature data for PPh_3_ ligands in coordination compounds) [34]. For complexes **9** and **5**, the Cl2p signal was also collected and analyzed, observing a single spin-orbit pair with the main Cl2p_3/2_ peak centered around 199.1 eV for **9** and a couple of signals (Cl2p_3/2_ BE = 199.2 eV and 201.3 eV, respectively) for **5**. While the lower BE signals are in good agreement with literature data about chlorine atoms coordinated to copper ions in coordination compounds with monomeric molecular structure (see Figure 2) [25,26,27,35], the C2p signal at higher BE values observed in **5** could suggest a Cu-Cl-Cu bridge, in which the double coordination at Cu(II) centers could be responsible for the observed BE shift towards more positive values for the chlorine atoms. This interpretation suggests that complex **9** is a monomer and complex **5** is a mixture of monomeric and dimeric structures.

### 2.3. Near Edge X-ray Absorption Fine Structure Studies: Functional Groups

Near edge X-ray absorption fine structure (NEXAFS) spectra were recorded at the C and N K-edges in order to gather further information about the chemical structure and stability of the Cu(II) coordination complexes.

**Figure 4:**
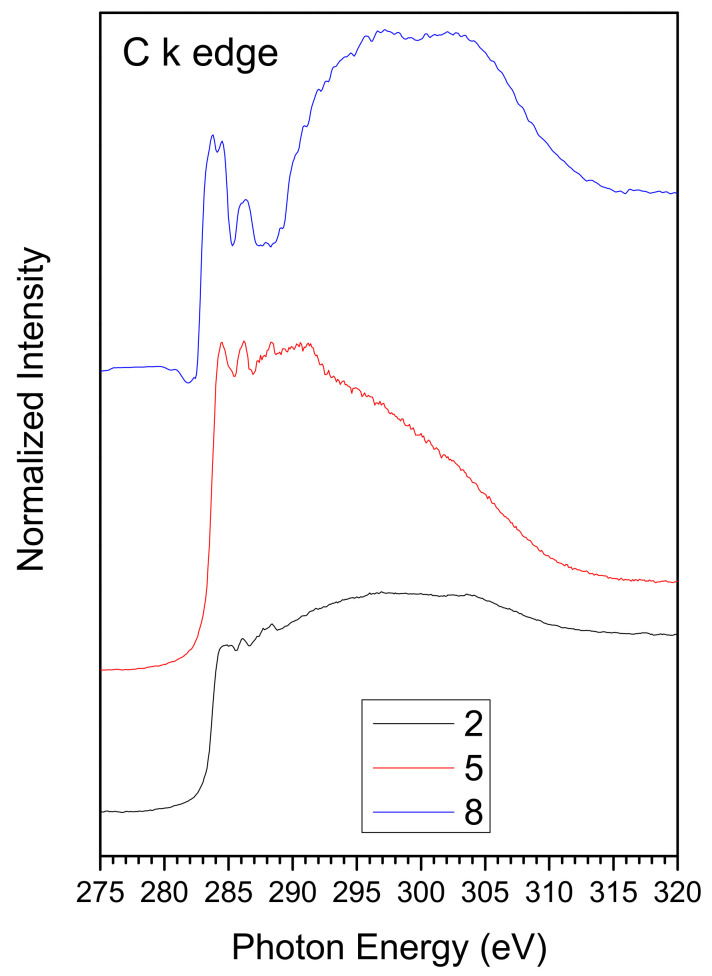

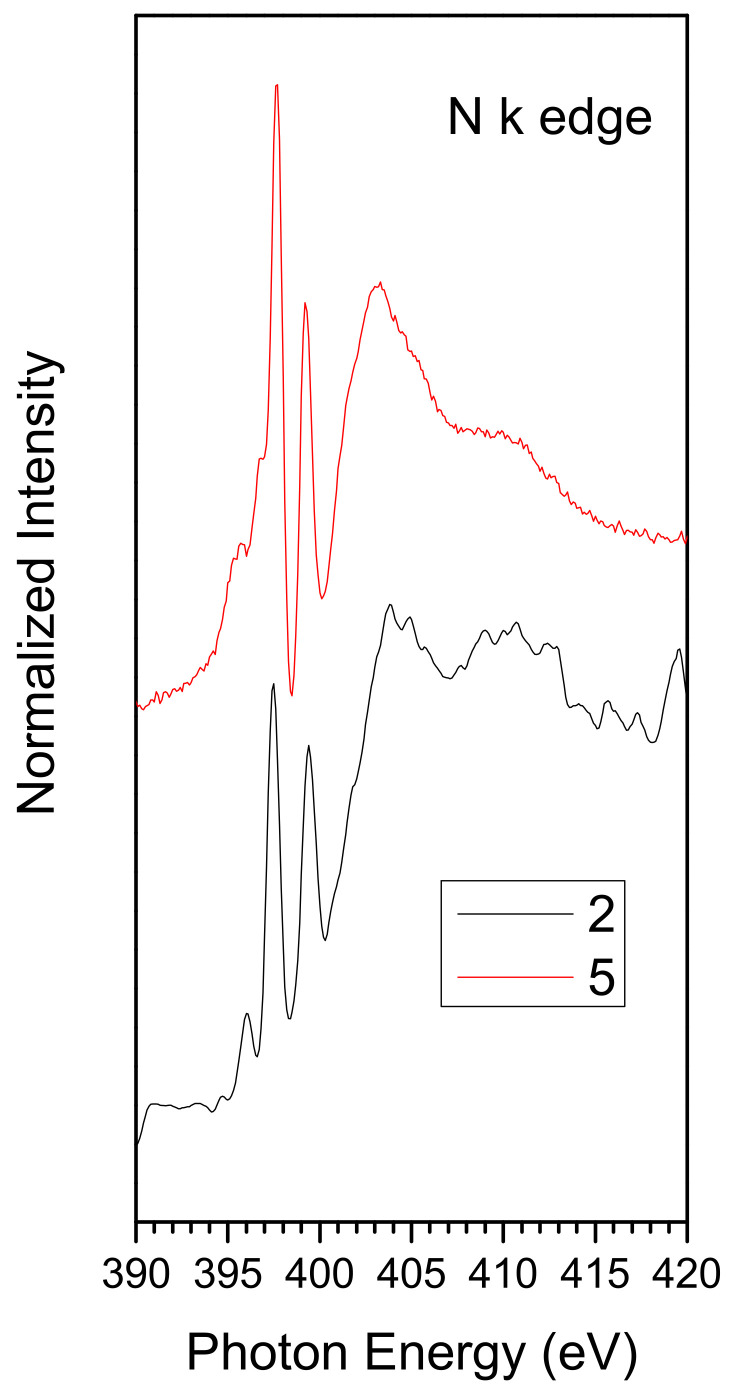
C K edge NEXAFS spectra of samples **2**, **5**, and **8** (**top**) and N K edge (**bottom**) NEXAFS spectra of samples **2** and **5**.

The spectra were collected at a grazing angle, i.e., with the photon beam impinging at 30° of incidence on the sample surface. C K spectra of samples **2**, **5**, and **8** and N K edge spectra of samples **2** and **5** are shown as an example in Figure 4; peak position and assignment of the main features detected in the spectra are shown in Table 1.

The energy scale referenced the $\;{\pi *}_{C=C}$ and ${\pi *}_{C=O}$ resonances for the C K edge spectra and to the π*_1_ resonance for the N K edge spectra.

In the C K edge spectra of samples **2** and **5**, C1s→π* transitions below the edge are due, respectively, to C=C (π*_C=C_) and C=N carbons (π*_C=C_) of the pyrazole rings and to the O-C=O carbon of the ester function (π*_C=O_), as already evidenced for similar complexes [25,26,27]. In the spectrum of sample **8**, the π*_C=C_ resonance is split in two peaks, related to C=C carbons of the pyrazole rings and of the benzene rings of triphenylphosphine. The two large features above the edge in the spectra of the three investigated samples are related to C1s→σ* transitions of singly bonded (σ*_C-C_) and doubly bonded carbons (σ*_C=N_).

In the N K edge spectra of samples **2** and **5**, two N1s→π* transitions (π*_1_ and π*_2_) related to the pyrazole rings were detected below the edge and two broad bands due to σ*_C=N_ and σ*_C-N_ resonances above the edge, as previously evidenced for similar complexes [25,26,27].

### 2.4. Coordination Geometry at the Copper Ion Site: XAS

**Figure 5:**
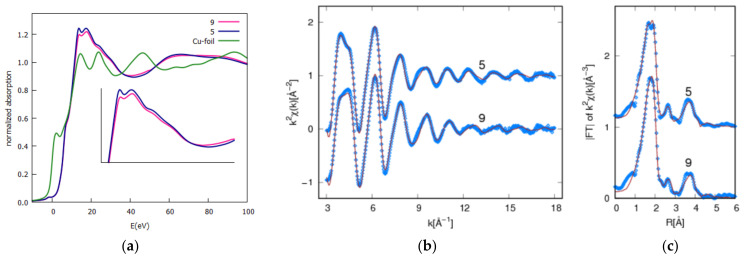
(**a**) Cu K edge normalized spectra in the near edge (XANES) region for complex **5**, **9**, and Cu reference metal foil. The spectra of **5** and **9** depict weak but evident differences in the XANES region (inset panel a); (**b**) $k^{2}$-weighted experimental (blue dots) EXAFS data and best fit (brown lines) curves for complexes **9** and **5** (vertically shifted for clarity); (**c**) moduli of $k^{2}\chi \left(k\right)$ Fourier transforms (|FT|) of experimental (blue dots) and best fit (brown lines) curves. The |FT| maxima represent the main neighbor coordination distances (uncorrected for the phase shift).

**Figure 6:**
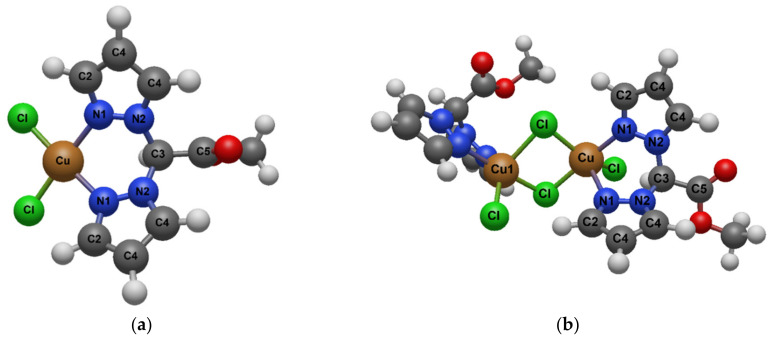
DFT models of the local atomic structure around Cu in complex **5** with both monomer (**a**) and dimer (**b**) configuration. For sake of XAFS data analysis, the dimer configuration was obtained by adding the Cu_1_ neighbor. Neighbor atoms are labeled to be individuated in the shell structure reported in Table 2.

X-ray absorption spectroscopy (XAS) measurements were carried out at the Cu K-edge on Cu(II) coordination compounds **5** and **9** (Figure 5) with the aim to probe the local atomic structure around the copper ion; more in detail, XAS data analysis was applied to disambiguate the monomeric or dimeric chemical structures, as depicted in Figure 6.

The Cu K edge XAS spectra in the near edge (XANES) region are shown in Figure 5a for sake of comparison. The XANES spectra of coordination compounds **5** and **9** rose at the same energy, pointing out the very same valence state in the two complexes but depicting differences in the XANES region (inset, Figure 5a), suggesting fine differences in the coordination chemistry of Cu ions in the two complexes.

The quantitative analysis of the EXAFS signals was carried out, fitting the $k^{2}$-weighted theoretical curves $k^{2}\chi_{th}\left(k\right)$ to the raw experimental spectra $k^{2}\chi_{exp}\left(k\right)$ in the 3–18 ${Å}^{-1}$ k range (without Fourier filtering or interpolation). The theoretical curves $\chi_{th}\left(k\right)$ were calculated as a sum of partial contributions $\chi_{i}$, considering selected single (SS) and multiple (MS) scattering contributions: $\chi_{th}\left(k\right)=\sum_{i}\chi_{i}$ along the lines described in [25]. The $\chi_{i}$ were calculated using the standard EXAFS formula [36,37] with Gaussian disorder approximation, and the theoretical photoelectron scattering amplitude and phase functions were calculated using FEFF8 program [38]. The factor $R_{w}^{2}=\frac{\sum_{k}{\left[k^{2}\left(\chi_{exp}-\chi_{th}\right)\right]}^{2}\;}{\sum_{k}{\left[k^{2}\chi_{exp}\right]}^{2}}$ was used to evaluate the best fit quality. The experimental data and best fit are presented in Figure 5b,c.

For accurate quantitative EXAFS analysis, it is of primary importance to have a reasonable model of the average coordination geometry around the absorber in order to select the relevant contributions ($\chi_{i}$) and to calculate the amplitude and phase function. Therefore, we calculated a plausible atomic model for Cu coordination using DFT, as described in the “Materials and Methods” section. The DFT model proposed for coordination compounds **5** and **9** in a monomeric configuration is reported in Figure 6. The DFT dimer model envisages a Cu next neighbor linked to the Cu absorber through the two Cl ions.

Based on the DFT model, we individuated the main single and multiple scattering contribution, grouped together those contributions having similar path length and type of neighbors (nitrogen and carbon give very similar EXAFS signals), and after a trial and error procedure aimed at minimizing the number of contributions and free parameters in the fitting, we found 4 SS (Cu-N_1_, Cu-Cl, Cu-C_2_/N_2_, and Cu-C_4_) and 2 MS (Cu-N_1_-C_2_ and Cu-N_1_/C_2_-C_4_) suitable to refine the experimental EXAFS data, taking into account the Cu local atomic structure in complexes until around 4.7 Å. To test for the possibility of dimer formation, we tried to improve the fit by adding a Cu-Cu_1_ contribution expected around 3.5 Å (Figure 6). We found that for complex **9**, the $R_{w}^{2}$ increased by about 20% (1.33 × 10^−2^ to 1.58 × 10^−2^) upon considering the Cu-Cu_1_ shell, unlike the presence of Cu dimers in **9**. On the contrary, the $R_{w}^{2}$ decreased by about 10% (from 1.27 × 10^−2^ to 1.12 × 10^−2^) in complex **5**, and the multiplicity number for Cu-Cu_1_ was found around 0.5(1). This finding suggests about 50% of Cu forming dimers in complex **5**. These findings are in excellent agreement with XPS Cl2p data, suggesting the presence of two different Cl atoms in complex **5** and a single spin orbit pair in **9**.

### 2.5. Stability Studies

**Figure 7:**
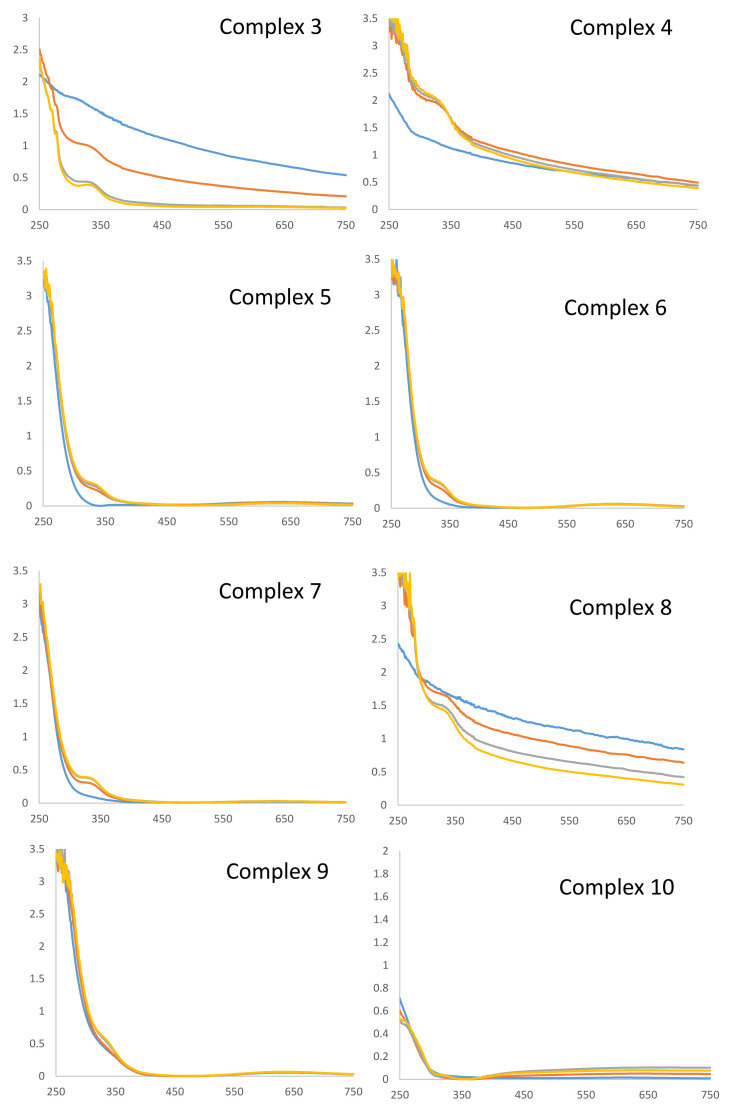
Stability studies. All complexes were dissolved at 50 μM in 0.5% DMSO/RPMI. UV-Vis spectra were recorded at t = 0 min (yellow), t = 24 h (grey), t = 48 (red), and t = 72 h (blue), at 37 °C.

The stability of the new complexes in 0.5% DMSO/RPMI (without phenol red) cell culture medium was also evaluated by UV-Vis spectroscopy. Samples were kept at 37 °C for the entire duration of the experiment, and spectra were collected every 24 h in the range of 750–250 nm over 72 h. The collected spectra for all the complexes are reported in Figure 7, and their comparison showed that all compounds were sufficiently stable in cell culture medium solutions.

In particular, among Cu(II) complexes, **9**–**10** were completely stable over 72 h, while complexes **5**–**6** proved complete stability at least up to 48 h. Concerning the Cu(I) species, complex **4** and **7** showed almost complete stability in cell culture medium (no evident change in their spectra up to 48 h). On the contrary, the spectra registered for complexes **3** and **8** showed curves with a progressive increase in the baseline adsorption, probably due to solubility issues. In particular, complex **3** proved to be the least stable compound in 0.5% DMSO/RPMI solution.

### 2.6. Cytotoxicity Studies

All Cu(I) and Cu(II) complexes **3**–**10** and the corresponding uncoordinated ligands **1**–**2** were evaluated for their cytotoxic activity against human cancer cell lines derived from different solid tumors. In particular, the in-house cancer cell panel contained examples of pancreatic (PSN-1), colon (HCT-15), ovarian (2008), lung small cell, and cervical (A431) cancers. For comparison purposes, cisplatin efficacy was assessed under the same experimental conditions. The cytotoxicity parameters, expressed in terms of IC_50_ and obtained after 72 h of drug exposure by MTT assay, are reported in Table 3.

Ligands **1**–**2** were proven to be completely ineffective in determining a reduction in cancer cell viability (IC_50_ values > 50 μM). On the contrary, all tested complexes **3**–**10** showed a very promising cytotoxic activity, with IC_50_ values in the micromolar range over the all-tested cell lines and being particularly effective against colon HCT-15 cancer cells. Compared with reference chemotherapeutic drug cisplatin, Cu(I) complexes **3**–**4** and **7**–**8** showed a comparable or lower cytotoxic potency, whereas Cu(II) complexes **5**–**6** and **9**–**10** were all significantly much more effective against tested cancer cell lines, with IC_50_ values in the sub-micromolar range. Among all, complex **3** was the weakest in reducing cancer cell proliferation, exhibiting an average IC_50_ value of 20 µM towards tested cell lines. It is noteworthy that complex **3** was the least stable in cell culture medium, and hence, its lower cytotoxic effectiveness could be attributed at least in part to its instability in physiological conditions. Conversely, complexes **9** and **10** were those showing the most effective in vitro antitumor potential, with average IC_50_ values of 0.3 and 0.6 µM, respectively. Again, stability shown by complexes **9**–**10** over 72 h seemed to well-correlate with their prominent cytotoxicity in 2D cell studies. Trying to draw some structure–activity relationships (SARs), cytotoxicity results indicated that Cu(II) complexes **5**–**6** and **9**–**10** were on average much more effective than the corresponding Cu(I) complexes **3**–**4** and **7**–**8**. Among Cu(II) derivatives, complexes **9**–**10**, bearing the ligand **2** with 3,5-dimethyl-pyrazoles, were on average about 5 and 2 times more effective with respect to compounds **5**–**6** bearing the unsubstituted pyrazole-based ligand, respectively. Coordination of Br or Cl to the metal center seemed to minimally influence cytotoxic activity. Similar to that seen with Cu(II) complexes, Cu(I) derivatives containing the 3,5-dimethyl-pyrazole ligand, **7**–**8**, were much more effective with respect to those bearing the unsubstituted ones, **3**–**4**. In addition, Cu(I) compounds with the more lipophilic PPh_3_ phosphane ligand, **4** and **8**, were mainly more effective than the corresponding PTA complexes **3** and **7**. It is interesting to note that overall, compounds showing a greater stability or solubility in cell culture medium were those endowed with the best in vitro antiproliferative activity, thus suggesting than chemical stability plays a crucial role in modulating the cytotoxicity profiles of Cu(I) and Cu(II) complexes.

Promoted by these very encouraging results, we also assessed the activity of the newly developed Cu(I) and Cu(II) complexes towards a cisplatin-resistant subline, the C13* human ovarian cancer cells. Actually, drug resistance represents a key determinant for the variable efficacy of anticancer therapy, and platinum-based resistance represents one of the major causes of failure of chemotherapeutic treatment. C13* cells were derived from the parent 2008 cells by 13 monthly selections to cisplatin exposure. In these cancer cells, the resistance mechanisms were detailed and consist of altered cellular drug uptake and higher intracellular thiols and glutathione and thioredoxin reductase levels, as well as enhanced repair of DNA damage [39].

Cross-resistance profiles were estimated by means of the resistance factor (RF), which is defined as the ratio among IC_50_ values obtained in resistant C13*cells and those calculated in the sensitive 2008 ones (Table 3). Remarkably, all tested compounds showed a very similar cytotoxic profile both in cisplatin-sensitive and -resistant cell lines. The RF values calculated for all derivatives were 3.7- to 37-fold lower than that of cisplatin, clearly indicating the absence of cross-resistance.

All tested compounds were also screened against 3D spheroids of human colon HCT-15 cancer cells in order to further assess their anticancer potential in a more predictive environment. Actually, 3D cell cultures possess several features that more closely resemble the complexity of the in vivo tumor architecture and physiology, being accordingly more predictive of the in vivo efficiency [40]. The cancer spheroids were treated with tested complexes for 72 h, and the cell viability was estimated by means of a modified acid phosphatase (APH) assay (Table 4).

Notably, all complexes were much more effective than cisplatin against the three-dimensional model. However, different from results obtained with 2D cell cultures, in 3D models, some Cu(I) complexes proved to be much more effective than Cu(II) derivatives. In particular, Cu(I) complexes **4** and **8** containing the PPh_3_ ligand in the copper coordination sphere emerged as the most promising derivatives, with IC_50_ values nearly 5- and 9-fold better than that of cisplatin, respectively.

These results could be related to the higher lipophilic character of the PPh_3_ ligand which can confer the entire Cu(I) complex a higher lipophilic character, thus making it more effective in penetrating across the entire spheroid domain and reaching the inner hypoxic core.

### 2.7. Cellular Uptake

**Figure 8:**
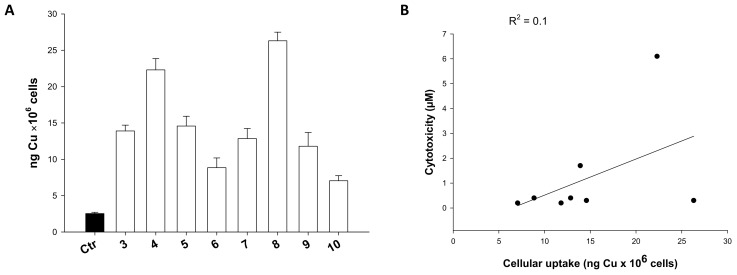
Cellular uptake (**A**) and correlation between cytotoxicity and cellular copper levels (**B**) in drug-treated HCT-15 colon cancer cells. Cells were incubated for 24 h with 0.75 µM of tested complexes. The amount of cellular Cu was estimated by GF-AAS. Error bars are S.D.

It has been extensively shown that one of the key factors determining the biological activity of a metal-based compound is its ability to enter cancer cells. Consequently, with the aim of correlating the antiproliferative effects elicited by the tested complexes and their cellular accumulation, Cu content was evaluated in HCT-15 cells treated with equal doses (0.75 μM) of the tested compounds for 24 h. The intracellular amounts of metal were quantified by means of the graphite furnace atomic absorption spectroscopy (GF-AAS), and the results, expressed as ng of metal per 10^6^ cells, are depicted in Figure 8, panel A. Although to a different extent, all tested complexes were able to cross cell membranes and to accumulate in HCT-15 cancer cells. In particular, derivatives **4** and **8** were those most efficiently internalized. By contrast, **6** and **10** gave the lowest copper accumulation in treated cells, with cellular uptake levels about three times lower compared to compound **8**. Interestingly, no linear correlation was found between the cytotoxic activity and the cellular uptake of copper compounds (Figure 8, panel B), as indicated by the very low R^2^ value.

However, some considerations can be drawn considering the different lipophilic character of coordinating coligands. Actually, the most internalized complexes were those bearing the higher hydrophobic PPh_3_ coligand, whereas bromide Cu(II) complexes **6** and **10** were those less able to accumulate in colon cancer cells. Complexes **3** and **7** bearing the more hydrophilic PTA phosphane ligand were both poorly accumulated into cancer cells.

It is also important to notice that complexes showing a better uptake profile were those endowed with the highest efficacy in 3D cell viability assays, thus suggesting that even if a direct correlation could not be found for all Cu complexes series, complexes that more efficiently accumulated in cancer cells were those more effective in permeating cancer spheroids.

### 2.8. Mechanistic Studies

During the past three decades, a number of research studies have been devoted to the elucidation of the mechanism of action of copper complexes. Several different molecular targets have been proposed for copper complexes, and a fine analysis of the literature allows speculation that the difference in redox state, donor atom set, and coordination geometries of copper species, besides defining their chemical reactivity, originates their different pharmacodynamic properties, thus consequently tailoring their biological activity and cancer cell selectivity [11,41,42].

We recently reported detailed investigations on the mechanism of action of some thiosemicarbazone (TSC) copper(II) complexes regarded as potent inhibitors of the protein disulfide isomerase (PDI), a copper-binding protein, that is emerging as a new therapeutic target for cancer treatment [43]. Afterwards, other research groups such as Heffeter and co-workers have reported that PDI could be a possible target for copper(II) [44,45].

**Figure 9:**
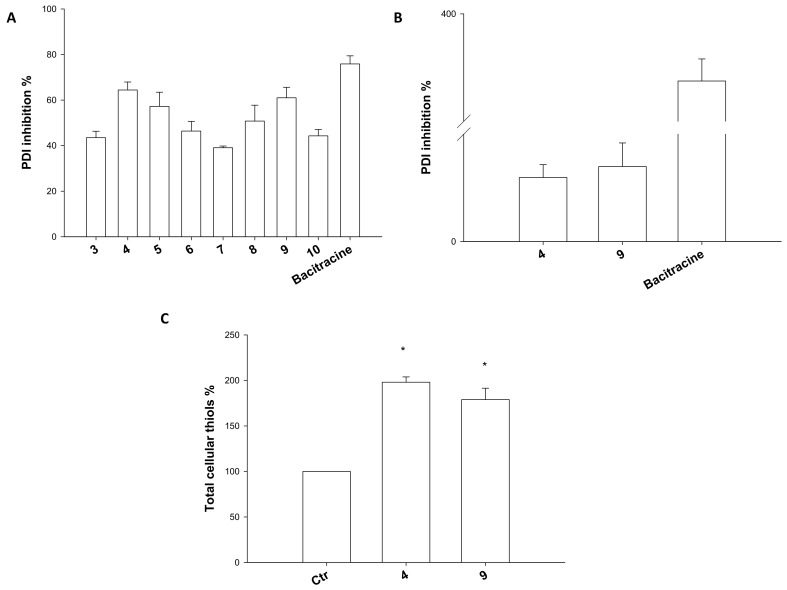
Mechanistic studies: PDI inhibition (**A**,**B**) and sulfhydryl content (**C**). (**A**,**B**) PDI inhibition induced by compounds **3**–**10** was measured by Proteostat PDI assay kit. The PDI inhibitor bacitracin (0.5 mM) was used as a positive control. In (**B**), the IC_50_ values were calculated by 4-PL model. (**C**) Sulfhydryl content in HCT15 cancer cells incubated for 36 h with IC_50_ of compounds **4** and **9**. The sulfhydryl group amount was determined by the DTNB assay. Error bars indicate S.D. * *p* < 0.05 compared with control.

On this basis, we thought it was of interest to evaluate the ability of our complexes to act as inhibitors of PDI reductase activity. The enzyme was treated with 5 µM of tested complexes, and the ability to hamper its activity was assessed by a biochemical colorimetric method (Proteostat kit). As reported in Figure 9, panel A, all complexes were effective in modulating PDI activity, and in particular, the majority of the complexes determined a reduction of more than 50% of enzyme activity. Among all, complexes **4** and **9** were the most effective inhibitors, with IC_50_ about 126 and 110 times lower compared to that of the well-known inhibitor bacitracin, respectively (Figure 9, panel B). On the contrary, complexes **3**, **7**, and **10** were those endowed with the lower inhibitory capacity. Concerning SARs, it is evident from the results that there were no significant differences among Cu(I) and Cu(II) complexes in the PDI inhibition pattern, and both Cu(I) and Cu(II) complexes were effective in targeting this enzyme.

As it is well-known the principal PDI function is to catalyze the reduction of disulfide bonds and the oxidation of thiols [46], we evaluated the levels of reduced thiols in colon HCT-15 cells treated with complexes **4** and **9**. Interestingly, cells treated for 36 h with IC_50_ of tested complexes showed an outstanding increase in total sulfhydryl content, thus letting us to assume that copper complexes can effectively target PDI also in intact cancer cells.

**Figure 10:**
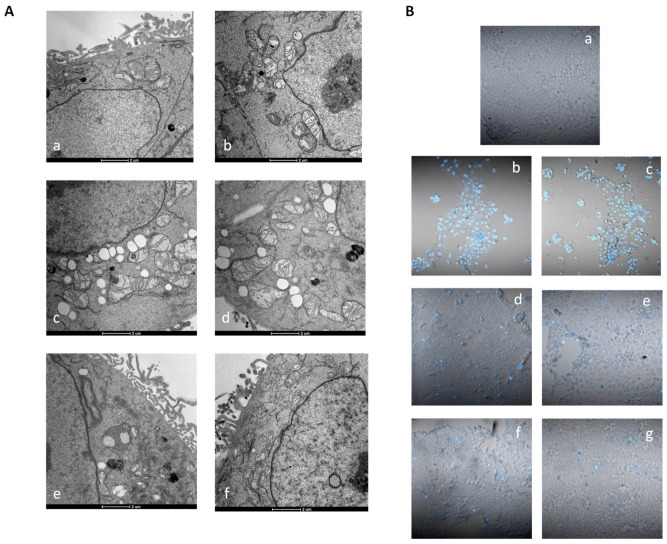
Morphological analysis (**A**) and cell death induction studies (**B**); (**A**) TEM analysis of HCT-15 colon cancer cells: (**a**,**b**) control cells; (**c**,**d**) HCT-15 cells treated for 24 h with IC_50_ concentrations of **4**; (**e**,**f**) HCT-15 cells treated for 24 h with IC_50_ concentrations of **8**; (**B**) Hoechst staining of HCT-15 cells: (**a**) control cells; HCT-15 cells incubated for 24 or 48 h with IC_50_ doses of **4** (**b**,**c**), **8** (**d**,**e**), or cisplatin (**f**,**g**).

PDI is an abundant endoplasmic reticulum (ER)-resident oxidoreductase that, being implicated in disulfide bonds, plays a crucial role in protein folding. PDI modification and inhibition is known to increase protein misfolding and to induce ER stress, ultimately leading to cell death [47]. Based on the above results, we performed electron microscopy analysis of HCT-15 cells in order to evaluate any direct morphological modification induced by treatment with tested complexes and corroborate the hypothesis that the newly developed Cu(I) and Cu(II) complexes act as PDI inhibitors in cancer cells (Figure 10, panel A).

Intriguingly, both Cu(I) complex **4** and Cu(II) derivative **9** showed to induce a massive induction of ER cisternae enlargement, which is a clear sign of ER stress, and a concomitant induction of cytoplasmic vacuolization. In addition, morphological analysis revealed that both complexes induced a slight increase in mitochondrial dimension (swelling) associated with a decreased electron density of the inner membrane and matrix regions and alteration of cristae features. However, mitochondria swelling was not consistent with cellular ROS induction. Actually, evaluation of effects induced by **4** and **9** on colon HCT-15 cancer cells in terms of cellular ROS production clearly showed that these complexes did not provoke any increase in cellular basal ROS levels (Figure S2).

Finally, as it has been widely described that Cu(I) and Cu(II) complexes can induce different types of cell death, namely apoptosis and paraptosis [11], we assessed the ability of selected **4** and **9** complexes to induce cancer cell death by means of apoptosis. Figure 10, panel B, shows the results obtained upon monitoring cellular morphological changes in HCT-15 cancer cells treated for 24 or 48 h with IC_50_ doses of **4** and **9** and stained with Hoechst 33,258 fluorescent probe. As expected, compared with control cells, cells treated with cisplatin presented brightly stained nuclei and morphological features typical of cells undergoing apoptosis, such as chromatin condensation and fragmentation. On the contrary, cells treated with both copper complexes for 24 or 48 h did not show any classical sign of apoptosis induction.

## 3. Materials and Methods

### 3.1. Chemistry

#### 3.1.1. Materials and General Methods

All reagents were purchased and used without further purification. All solvents were dried, degassed, and distilled prior to use. Melting points were performed by an SMP3 Stuart Scientific Instrument (Bibby Sterilin Ltd., London, UK). Elemental analyses (C, H, N, and S) were performed with a Fisons Instruments EA-1108 CHNS-O Elemental Analyzer (Thermo Fisher Scientific Inc., Waltham, MA, USA). Fourier-transform infrared (FT-IR) spectra were recorded from 4000 to 200 cm^−1^ on a PerkinElmer Frontier Instrument (PerkinElmer Inc., Waltham, MA, USA), equipped with attenuated total reflection (ATR) unit using a universal diamond top-plate as the sample holder. Abbreviation used in the analyses of the FT-IR spectra are: m = medium, mbr = medium broad, s = strong, sbr = strong broad, sh = shoulder, vs = very strong, w = weak, and wbr = weak broad. ^1^H-, ^13^C-, and ^31^P-NMR spectra were recorded with a Bruker 500 Ascend Spectrometer (Bruker BioSpin Corporation, Billerica, MA, USA; 500.1 MHz for ^1^H, 125 MHz for ^13^C, and 202.4 MHz for ^31^P). Referencing was relative to tetramethylsilane (TMS) (^1^H and ^13^C) and 85% H_3_PO_4_ (^31^P). NMR annotations used are as follows: br = broad, d = doublet, dbr = doublet broad m = multiplet, mbr = multiplet broad, s = singlet, sbr = singlet broad, sept = septet, and t = triplet. Electrospray ionization mass spectra (ESI-MS) were recorded in positive- (ESI-MS(+)) or negative-ions (ESI-MS(−)) mode on an Waters Micromass ZQ Spectrometer equipped with a single quadrupole (Waters Corporation, Milford, MA, USA), using a methanol or acetonitrile mobile phase. The compounds were added to reagent grade methanol or acetonitrile to give approximately 0.1 mM solutions. These solutions were injected (1 µL) into the spectrometer fitted with an autosampler. The pump delivered the solutions to the mass spectrometer source at a flow rate of 200 μL/min, and nitrogen was employed both as a drying and nebulizing gas. Capillary voltage was typically 2500 V. The temperature of the source was 100°C, while the temperature of the desolvation was 400 °C. In the analyses of ESI-MS spectra, the confirmation of major peaks was supported by comparison of the observed and predicted isotope distribution patterns, the latter calculated using the IsoPro 3.1 computer software (T-Tech Inc., Norcross, GA, USA).

The precursors HC(pz)_2_COOH (LH) [48] and HC(pz^Me2^)_2_COOH (L^2^H) [49] were prepared by the literature method. The ligands [HC(pz)_2_COOCH_3_] (L^OMe^, **1**) [50] and [HC(pz^Me2^)_2_COOCH_3_] (L^2OMe^, **2**) were prepared by the literature method [51] and were fully characterized (^1^H-NMR: Figures S3 and S4).

#### 3.1.2. Synthesis of [(PTA)Cu(L^OMe^)]PF_6_ (**3**)

[Cu(CH_3_CN)_4_]PF_6_ (0.600 mmol, 0.224 g) was added to a solution of 1,3,5-triaza-7-phosphaadamantane (PTA, 0.600 mmol, 0.095 g) in acetonitrile (50 mL). The reaction mixture was stirred at room temperature for 3 h; then, ligand **1** (0.600 mmol, 0.124 g) was added, and the suspension was stirred for 24 h at room temperature. The reaction mixture was filtered, and the mother liquors were dried at reduced pressure to give the whitish complex [(PTA)Cu(L^OMe^)]PF_6_ (**3**) in 81% yield. M.p.: 180–185 °C. FT-IR (cm^−1^): 3135 wbr, 2954 wbr (C-H); 1759 m (C=O); 1654 wbr; 1519 w (C=C/C=N); 1451 wbr, 1416 sh, 1403 m, 1395 wbr, 1353 wbr, 1295 mbr, 1238 mbr, 1206 w, 1179 w, 1099 m, 1057 w, 1039 w, 1017 m, 970 m, 950 m, 918 w, 895 wbr; 831 vs (PF_6_^−^); 817 sh, 758 s, 741 sh, 655 m, 610 m; 555 vs (PF_6_^−^); 445 m, 396 m. ^1^H-NMR (CD_3_CN, 293 K, Figure S5): δ 3.78 (s, 3H, OC*H*_3_), 4.09 (s, 6H, NC*H*_2_P), 4.48–4.57 (m, 6H, NC*H*_2_N), 6.44 (t, 2H, 4-C*H*), 7.32 (s, 1H, C*H*CO), 7.66 (d, 2H, 5-C*H*), 7.92 (d, 2H, 3-C*H*). ^13^C{^1^H}-NMR (CD_3_CN, 293 K): δ 49.89, 53.65, 72.49, 73.07, 106.97, 132.11, 141.86, 164.73. ^31^P{^1^H}-NMR (CD_3_CN, 293 K): δ −93.52 (sbr), −143.51 (sept, J_(P-F)_ = 708 Hz, PF_6_). ^31^P{^1^H}-NMR (CD_3_CN, 243 K): δ −95.28 (sbr), −144.75 (sept, J_(P-F)_ = 707 Hz, PF_6_). ESI-MS (major positive ions, CH_3_CN), *m*/*z* (%): 158 (65) [PTA + H]^+^, 269 (100) [Cu(L^OMe^)]^+^, 426 (20) [(PTA)Cu(L^OMe^)]^+^, 475 (10) [Cu(L^OMe^)_2_]^+^. ESI-MS (major negative ions, CH_3_CN), *m*/*z* (%): 145 (100) [PF_6_]^−^. Elemental analysis (%) calculated for C_15_H_22_CuF_6_N_7_O_2_P_2_: C 31.50, H 3.88, N 17.15; found: C 31.56, H 3.68, N 16.86.

#### 3.1.3. Synthesis of [(PPh_3_)Cu(L^OMe^)]PF_6_ (**4**)

This compound was prepared following the procedure described for **3**, using triphenylphosphine (PPh_3_, 0.600 mmol, 0.157 g), to give the whitish complex [(PPh_3_)Cu(L^OMe^)]PF_6_ (**4**) in 74% yield. M.p.: 60–65 °C. FT-IR (cm^−1^): 3137 wbr, 3055 wbr, 3006 wbr, 2959 w, 2918 w, 2850 w (C-H); 1760 m (C=O); 1523 wbr, 1481 m (C=C/C=N); 1454 m, 1436 m, 1403 m, 1376 w, 1356 w, 1307 sh, 1295 mbr, 1227 m, 1207 w, 1180 w, 1161 w, 1097 s, 1058 m, 1027 w, 998 m, 982 sh, 974 m, 921 w, 872 sh; 830 vs (PF_6_^−^); 760 sh, 744 vs, 693 s, 610 m; 556 s (PF_6_^−^); 531 s, 504 s, 430 m, 334 m, 280 m, 252 m, 218 m. ^1^H-NMR (CD_3_CN, 293 K, Figure S6): δ 3.65 (s, 3H, OC*H*_3_), 6.42 (t, 2H, 4-C*H*), 7.34-7.53 (m, 16H, Ar*H* and C*H*CO), 7.59 (d, 2H, 5-C*H*), 7.93 (d, 2H, 3-C*H*). ^13^C{^1^H}-NMR (CD_3_CN, 293 K): δ 53.51, 73.26, 106.99, 128.92, 130.36, 132.00, 132.40, 132.66, 133.42, 141.75, 164.80. ^31^P{^1^H}-NMR (CD_3_CN, 293 K): δ −1.48 (sbr), −144.62 (sept, J_(P-F)_ = 707 Hz, PF_6_). ESI-MS (major positive ions, CH_3_CN), *m*/*z* (%): 269 (15) [Cu(L^OMe^)]^+^, 531 (100) [(PPh_3_)Cu(L^OMe^)]^+^, 587 (65) [(PPh_3_)_2_Cu]^+^. ESI-MS (major negative ions, CH_3_CN), *m*/*z* (%): 145 (100) [PF_6_]^−^. Elemental analysis (%) calculated for C_27_H_25_CuF_6_N_4_O_2_P_2_: C 47.90, H 3.72, N 8.28; found: C 48.45, H 3.77, N 7.96.

#### 3.1.4. Synthesis of [(L^OMe^)CuCl_2_] (**5**)

Ligand **1** (1.000 mmol, 0.206 g) was added to a CuCl_2_·2H_2_O (1.000 mmol, 0.170 g) solution in CH_3_OH (50 mL). The reaction was carried out under magnetic stirring for 24 h at room temperature. Then, the suspension was filtered, and the precipitate was dried at reduced pressure to give the light green complex [(L^OMe^)CuCl_2_] (**5**) in 75% yield. M.p.: 208–212 °C. FT-IR (cm^−1^): 3139 w, 3117 w, 2990 wbr, 2959 wbr (C-H); 1748 vs (C=O); 1518 w, 1510 w (C=C/C=N); 1454 m, 1431 m, 1415 m, 1403 s, 1355 w, 1293 sh, 1285 vs, 1255 m, 1230 s, 1198 m, 1176 m, 1148 w, 1105 m, 1093 m, 1072 s, 1059 s, 1003 m, 989 m, 973 s, 924 w, 911 w, 869 w, 854 m, 805 m, 771 vs, 654 m, 607 s, 597 s, 400 m, 352 m, 342 m; 305 s; 278 vs (Cu-Cl); 223 m. ESI-MS (major positive ions, CH_3_ OH), *m*/*z* (%): 206 (50) [L^OMe^ + H]^+^, 229 (100) [L^OMe^ + Na]^+^, 269 (55) [(L^OMe^ − H)Cu]^+^, 475 (20) [(L^OMe^)Cu(L^OMe^ − H)]^+^. ESI-MS (major negative ions, CH_3_OH), *m*/*z* (%): 170 (100) [CuCl_3_]^−^. Elemental analysis (%) calculated for C_9_H_10_Cl_2_CuN_4_O_2_: C 31.73, H 2.96, N 16.45; found: C 31.79, H 2.66, N 16.63.

#### 3.1.5. Synthesis of [(L^OMe^)CuBr_2_] (**6**)

This compound was prepared following the procedure described for **5**, using CuBr_2_ (1.000 mmol, 0.223 g), to give the reddish-brown complex [(L^OMe^)CuBr_2_] (**6**) in 60% yield. M.p.: 192–195 °C. FT-IR (cm^−1^): 3147 w, 3133 w, 3119 m, 2979 wbr, 2956 wbr (C-H); 1748 s (C=O); 1515 wbr (C=C/C=N); 1454 m, 1430 m, 1412 m, 1400 s, 1354 w, 1296 sh, 1283 s, 1254 m, 1226 s, 1197 m, 1175 m, 1148 wbr, 1105 m, 1091 m, 1071 m, 1058 s, 1003 m, 987 m, 972 sbr, 922 wbr, 907 m, 862 w, 852 m, 806 m, 765 vs, 651 m, 605 s, 595 s, 398 m, 341 m, 306 m; 235 vs (Cu-Br); 202 m. ESI-MS (major positive ions, CH_3_OH), *m*/*z* (%): 229 (100) [L^OMe^ + Na]^+^, 269 (60) [(L^OMe^ − H)Cu]^+^, 350 (85) [(L^OMe^)CuBr]^+^. ESI-MS (major negative ions, CH_3_OH), *m*/*z* (%): 304 (100) [CuBr_3_]^−^. Elemental analysis (%) calculated for C_9_H_10_Br_2_CuN_4_O_2_: C 25.16, H 2.35, N 13.04; found: C 25.35, H 2.27, N 12.59.

#### 3.1.6. Synthesis of [(PTA)Cu(L^2OMe^)]PF_6_ (**7**)

This compound was prepared following the procedure described for **3**, using the ligand **2** (0.600 mmol, 0.157 g), to give the whitish complex [(PTA)Cu(L^2OMe^)]PF_6_ (**7**) in 86% yield. M.p.: 189–193 °C. FT-IR (cm^−1^): 3147 vw, 2954 sh, 2924 wbr (C-H); 1759 m (C=O); 1640 wbr; 1562 w (C=C/C=N); 1446 sh, 1420 m, 1392 w, 1317 m, 1285 w, 1270 w, 1242 m, 1230 m, 1155 sh, 1126 w, 1102 w, 1037 w, 1016 m, 972 m, 950 m, 895 w, 868 sh; 833 vs (PF_6_^−^); 742 m, 712 m; 556 s (PF_6_^−^); 453 m, 397 m, 297 m, 249 m, 223 m. ^1^H-NMR (CD_3_CN, 293 K, Figure S7): δ 2.27 (sbr, 6H, 3- or 5-C*H*_3_), 2.41 (sbr, 6H, 3- or 5-C*H*_3_), 3.76 (s, 3H, OC*H*_3_), 4.10 (s, 6H, NC*H*_2_P), 4.48-4.58 (m, 6H, NC*H*_2_N), 6.11 (sbr, 2H, 4-C*H*), 6.81 (s, 1H, C*H*CO). ^13^C{^1^H}-NMR (CD_3_CN, 293 K): δ 10.22, 13.25, 50.48, 53.81, 65.96, 72.59, 106.50, 143.43, 151.25, 164.75. ^31^P{^1^H}-NMR (CD_3_CN, 293 K): δ −95.72 (sbr), −144.78 (sept, J_(P-F)_ = 711 Hz, PF_6_). ESI-MS (major positive ions, CH_3_CN), *m*/*z* (%): 158 (5) [PTA + H]^+^, 263 (15) [L^2OMe^ + H]^+^, 285 (30) [L^2OMe^ + Na]^+^, 325 (20) [Cu(L^2OMe^)]^+^, 424 (15) [(PTA)Cu((pz^Me2^)_2_CH_2_)]^+^, 482 (100) [(PTA)Cu(L^2OMe^)]^+^, 529 (40) [(L^2OMe^)Cu((pz^Me2^)_2_CH_2_)]^+^, 587 (50) [Cu(L^2OMe^)_2_]^+^. ESI-MS (major negative ions, CH_3_CN), *m*/*z* (%): 145 (100) [PF_6_]^−^. Elemental analysis (%) calculated for C_19_H_30_CuF_6_N_7_O_2_P_2_: C 36.34, H 4.82, N 15.61; found: C 37.26, H 4.96, N 14.95.

#### 3.1.7. Synthesis of [(PPh_3_)Cu(L^2OMe^)]PF_6_ (**8**)

[Cu(CH_3_CN)_4_]PF_6_ (0.600 mmol, 0.224 g) was added to a solution of PPh_3_ (0.600 mmol, 0.157 g) in acetonitrile (30 mL). The reaction mixture was stirred at room temperature for 3 h; then, an acetonitrile solution of ligand **2** (0.600 mmol, 0.157 g) was added, and the suspension was stirred for 24 h at room temperature. The solution was dried at reduced pressure, and the residue was washed with Et_2_O to give the whitish complex [(PPh_3_)Cu(L^2OMe^)]PF_6_ (**8**) in 86% yield. M.p.: 199–204 °C. FT-IR (cm^−1^): 3145 vw, 3057 vw, 2994 vw, 2957 vw, 2923 vw (C-H); 1768 m (C=O); 1564 w (C=C/C=N); 1481 w, 1465 w, 1436 m, 1420 w, 1394 wbr, 1375 w, 1317 m, 1300 w, 1277 w, 1263 w, 1224 wbr, 1184 w, 1161 w, 1127 w, 1113 w, 1096 w, 1072 w, 1040 w, 1028 sh, 999 w, 877 sh; 836 vs (PF_6_^−^); 780 m, 747 m, 698 s, 636 w, 585 w; 557 s (PF_6_^−^); 527 s, 512 m, 491 m, 444 w, 363 w, 298 w, 279 m, 251 m, 229 w, 213 m. ^1^H-NMR (CD_3_CN, 293 K, Figure S8): δ 2.17 (s, 6H, 3- or 5-C*H*_3_), 2.47 (s, 6H, 3- or 5-C*H*_3_), 3.05 (sbr, 3H, OC*H*_3_), 6.19 (sbr, 2H, 4-C*H*), 6.91 (s, 1H, C*H*CO), 7.39–7.53 (m, 15H, Ar*H*). ^13^C{^1^H}-NMR (CD_3_CN, 293 K): δ 10.55, 13.64, 53.73, 106.99, 129.07, 130.50, 132.24, 133.43, 143.84, 151.70, 164.96. ^31^P{^1^H}-NMR (CD_3_CN, 293 K): δ −2.08 (sbr), −144.63 (sept, J_(P-F)_ = 706 Hz, PF_6_). ESI-MS (major positive ions, CH_3_CN), *m*/*z* (%): 285 (15) [L^2OMe^ + Na]^+^, 325 (15) [Cu(L^2OMe^)]^+^, 529 (40) [(L^2OMe^)Cu((pz^Me2^)_2_CH_2_)]^+^, 587 (100) [(PPh_3_)Cu(L^2OMe^)]^+^. ESI-MS (major negative ions, CH_3_CN), *m*/*z* (%): 145 (100) [PF_6_]^−^. Elemental analysis (%) calculated for C_31_H_33_CuF_6_N_4_O_2_P_2_: C 50.79, H 4.54, N 7.64; found: C 50.59, H 4.50, N 7.98.

#### 3.1.8. Synthesis of [(L^2OMe^)CuCl_2_] (**9**)

Ligand **2** (1.000 mmol, 0.262 g) was added to a CuCl_2_·2H_2_O (1.000 mmol, 0.170 g) solution in CH_3_OH (50 mL). The reaction was carried out under magnetic stirring for 24 h at room temperature. The solution was dried at reduced pressure, and the residue was washed with Et_2_O to give the orange complex [(L^2OMe^)CuCl_2_] (**9**) in 64% yield. M.p.: 179–182 °C. FT-IR (cm^−1^): 3143 w, 3105 wbr, 2972 w, 2955 w (C-H); 1761 m (C=O); 1645 wbr; 1561 m (C=C/C=N); 1489 sh, 1460 m, 1440 m, 1423 m, 1388 mbr, 1316 m, 1282 m, 1260 m, 1223 m, 1169 w, 1134 w, 1125 w, 1047 m, 997 sh, 985 m, 912 w, 902 m, 870 w, 846 m, 821 m, 784 m, 760 wbr, 722 m, 709 m, 662 w, 636 m, 582 w, 488 w, 365 m, 326 s; 279 vs (Cu-Cl); 228 m, 208 m. ESI-MS (major positive ions, CH_3_OH), *m*/*z* (%): 266 (25) [Cu((pz^Me2^)_2_CH)]^+^, 420 (30) [(L^2OMe^)CuCl_2_ + Na]^+^, 572 (100) [Cu(L^2OMe^)(L^2OMe^ − Me)]^+^. ESI-MS (major negative ions, CH_3_OH), *m*/*z* (%): 170 (100) [CuCl_3_]^−^. Elemental analysis (%) calculated for C_13_H_18_Cl_2_CuN_4_O_2_: C 39.35, H 4.57, N 14.12; found: C 39.44, H 4.58, N 13.78.

#### 3.1.9. Synthesis of [(L^2OMe^)CuBr_2_] (**10**)

This compound was prepared following the procedure described for **5**, using CuBr_2_ (1.000 mmol, 0.233 g) and ligand **2** (1.000 mmol, 0.262 g), to give the greenish-brown complex [(L^2OMe^)CuBr_2_] (**10**) in 51% yield. M.p.: 176–180 °C. FT-IR (cm^−1^): 3150 wbr, 2951 wbr, 2922 wbr (C-H); 1757 m (C=O); 1562 mbr (C=C/C=N); 1486 wbr, 1462 mbr, 1434 m, 1417 m, 1372 mbr, 1318 m, 1299 m, 1273 m, 1242 m, 1194 wbr, 1156 wbr, 1140 w, 1114 w, 1059 w, 1047 m, 1034 m, 985 mbr, 914 m, 903 m, 864 m, 814 s, 783 m, 721 m, 704 m, 634 m, 579 m, 487 m, 363 m, 305 m, 275 m, 253 s; 229 vs (Cu-Br). ESI-MS (major positive ions, CH_3_OH), *m*/*z* (%): 263 (50) [(L^2OMe^ + H)]^+^, 285 (100) [(L^2OMe^ + Na)]^+^, 572 (50) [Cu(L^2OMe^)(L^2OMe^ − Me)]^+^, 587 (80) [Cu(L^2OMe^)(L^2OMe^ − H)]^+^. ESI-MS (major negative ions, CH_3_OH), *m*/*z* (%): 304 (100) [CuBr_3_]^−^. Elemental analysis (%) calculated for C_13_H_18_Br_2_CuN_4_O_2_: C 32.15, H 3.74, N 11.54; found: C 32.16, H 3.77, N 11.39.

#### 3.1.10. Stability Studies in DMSO/RPMI

All complexes were dissolved at 50 μM in 0.5% DMSO/RPMI. UV-Vis spectra were recorded at t = 0 min, t = 24 h, t = 48, and t = 72 h by a Perkin-Elmer Lambda 25 (Perkin-Elmer).

### 3.2. Spectroscopic Methods

#### 3.2.1. Synchrotron Radiation (SR)-Induced X-ray Photoelectron Spectroscopy (SR-XPS)

Measurements were performed using the materials science beamline (MSB) at the ELETTRA synchrotron radiation source (Trieste, Italy) (Ceric proposal # 20192011). MSB was placed at the left end of the bending magnet 6.1, and it was equipped with a plane grating monochromator that provides light in the energy range of 21–1000 eV. The base pressure in the UHV end-station was of 2 × 10^−10^ mbar; the end-station was equipped with a SPECS PHOIBOS 150 hemispherical electron analyzer, low-energy electron diffraction optics, a dual-anode Mg/Al X-ray source, an ion gun, and a sample manipulator with a K-type thermocouple attached to the rear side of the sample. For this experiment, photoelectrons emitted by C1s, N1s, O1s, Cl2p, Cu2p, O1s, and P2p core levels were detected at normal emission geometry on powder (solid state) samples. We selected a photon energy value of 650 eV impinging at 60° to acquire all signals, with the aim to especially maximize N1s signal intensity. To collect Cu2p spectra, we also used the Al K-a anode source (1487.0 eV), allowing to maximize its photoemission signal. To calibrate binding energies (BEs), we used the aromatic C 1s as a reference (BE 284.70 eV) [52]. To fit core level spectra, we subtracted a polynomial background and then used Gaussian peak functions as signal components.

#### 3.2.2. Near Edge X-ray Absorption Fine Structure (NEXAFS)

Spectroscopy experiments were performed at the ELETTRA storage ring at the bending magnet for emission absorption and reflectivity (BEAR) beamline, installed at the left exit of the 8.1 bending magnet exit. The apparatus was based on a bending magnet as a source and beamline optics delivering photons from 5 eV up to about 1600 eV with selectable degree of ellipticity.

The carbon and nitrogen K-edge spectra were collected at a grazing incidence angle of the linearly polarized photon beam with respect to the sample surface.

The photon energy and resolution were calibrated and experimentally tested at the K absorption edges of Ar, N_2_, and Ne.

The normalization procedure consisted of three steps: (i) the energy calibration, in which the I_0_ reference current (drain current) of the sample is shifted on the I_0_ reference current (drain current) of the Au clean sample recorded; (ii) the signal is obtained by the double ratio I_sampleI0_sampleI_AuI0_Au after the interpolation of each signal to the Au reference, where the double ratio allows correcting for variations of the incident X-ray intensity as a function of photon energy due to instabilities of the electron beam in the storage ring or to changes of the X-ray optics in the beamline [53]; and finally, (iii) the signal is reduced to the standard form through a pre-edge and post-edge fit: a linear pre-edge background is subtracted from the data, and a linear post edge fit is applied to the post edge region to evaluate the jump and obtain the normalized signal.

#### 3.2.3. The Cu K Edge X-ray Absorption Spectroscopy (XAS)

Experiments were carried out at the 11.1R XAFS beamline [54] of the ELETTRA synchrotron radiation facility (Ceric proposal # 20192011), where the beamline optics was equipped with a double crystals (Si 311) monochromator and harmonic rejection mirrors. The Cu complex powders were dried in vacuum, mixed with graphite (1/10 weight ratio) and pressed to obtain a homogeneous pellet suitable for mounting on the sample holder. Cu K edge spectra were collected in transmission geometry in the 8650–10,400 eV range, two gas-filled ionization chambers were used to measure the incident (I_o_) and transmitted (I_1_) intensities, the XAS signal was calculated ($\alpha_{exp}=\ln\frac{I_{o}}{I_{1}}$), and 4 scans were collected and averaged up for each sample. Samples were kept in vacuum at liquid nitrogen (LN) temperature, and the graphite matrix ensured good thermal contact between the sample and cryostat cold finger. A Cu metal foil was placed in vacuum after the I_1_ chamber, and the transmitted intensity (I_2_) was measured using a third ionization chamber. The XAS signal of the reference Cu foil ($\alpha_{ref}=\ln\frac{I_{1}}{I_{2}}$) was used to carefully monitor the beamline energy calibration. The experimental spectra were treated along the standard procedures for background subtraction, edge jump normalization, and bare atom background subtraction [55] to extract the EXAFS structural signals $\chi_{exp}\left(k\right).$ The edge energy (*E_o_*) defines the energy scale of the photoelectron wavenumber *k* (being $k\;\left[{Å}^{-1}\right]=0.5123\sqrt{\left(E-E_{o}\right)}$ with energies in eV), and it was selected at the first inflection point of the Cu reference foil edge and refined (ΔE) during data fitting (see “Results and Discussion” section).

#### 3.2.4. Density Functional Theory (DFT) Calculations

In order to build the atomic cluster representing the Cu(II) coordination compounds **5** and **9**, we started with a model generated using a 3D open source software Avogadro followed by DFT calculation using the open source software ORCA 5.0.1 (using Becke ‘88 exchange and Perdew ‘86 correlation integrals within the energy functional). Karlsruhe orbital basis sets such as def2-SVP and def2-TZVP were used, and respectively a valence double zeta basis set for lighter atoms and a valence triple zeta basis for Cu atoms. Finally, the coordination compound structures were relaxed to a new minimum of energy using a Quasi-Newton method. In particular, Cu(II) ions showed a binding site described in Figure 6 for the two proposed structures, either monomeric or dimeric.

### 3.3. Experiments with Cultured Human Cancer Cells

Cu(I) and Cu(II) complexes and the corresponding uncoordinated ligands were dissolved in DMSO just before the experiment, and a calculated amount of drug solution was added to the cell growth medium to a final solvent concentration of 0.5%, which had no detectable effects on cell viability. Cisplatin (Sigma Chemical Co., St. Louis, MO, USA) (Sigma Chemical Co.) was dissolved in 0.9% sodium chloride solution.

#### 3.3.1. Cell Cultures

Human colon (HCT-15), pancreatic (PSN-1), and lung (U1285) carcinoma cell lines were obtained by American Type Culture Collection (ATCC, Rockville, MD, USA). A431 are human cervical carcinoma cells kindly provided by Professor F. Zunino (Division of Experimental Oncology B, Istituto Nazionale dei Tumori, Milan, Italy). The 2008 cells and cisplatin-resistant variant, C13*, are human ovarian adenocarcinoma cell lines that were kindly provided by Professor G. Marverti (Department of Biomedical Science of Modena University, Italy). Cell lines were maintained in the logarithmic phase at 37 °C in a 5% carbon dioxide atmosphere using RPMI-1640 medium (EuroClone) containing 10% fetal calf serum (EuroClone, Milan, Italy), antibiotics (50 units/mL penicillin and 50 μg/mL streptomycin), and 2 mM L-glutamine.

#### 3.3.2. MTT Assay

The growth inhibitory effect towards tumor cells was evaluated by means of MTT assay. Briefly, 3–8 × 10^3^ cells/well, dependent upon the growth characteristics of the cell line, were seeded in 96-well microplates in growth medium (100 μL). After 24 h, the medium was removed and replaced with a fresh one containing the compound to be studied at the appropriate concentration. Triplicate cultures were established for each treatment. After 72 h, each well was treated with 10 μL of a 5 mg/mL MTT saline solution, and following 5 h of incubation, 100 μL of a sodium dodecyl sulfate (SDS) solution in HCl 0.01 M was added. After an overnight incubation, cell growth inhibition was detected by measuring the absorbance of each well at 570 nm using a Bio-Rad 680 microplate reader. Mean absorbance for each drug dose was expressed as a percentage of the control untreated well absorbance and plotted vs. drug concentration. IC_50_ values, the drug concentrations that reduce the mean absorbance at 570 nm to 50% of those in the untreated control wells, were calculated by the four-parameter logistic (4-PL) model. Evaluation was based on means from at least four independent experiments.

#### 3.3.3. Spheroid Cultures

Spheroid cultures were obtained by seeding 2.5 × 10^3^ HCT-15 cancer cells/well in a round-bottom non-treated tissue culture 96-well plate (Greiner Bio-one, Kremsmünster, Austria) in phenol red-free RPMI-1640 medium (Sigma Chemical Co.) containing 10% fetal calf serum and supplemented with 20% methyl cellulose stock solution.

#### 3.3.4. Acid Phosphatase (APH) Assay

An APH modified assay was used for determining cell viability in 3D spheroids, as previously described [7]. Briefly, the pre-seeded spheroids were treated with fresh medium containing the compound to be studied at the appropriate concentration (range 5–150 μM). Triplicate cultures were established for each treatment. After 72 h, each well was treated with 100 μL of the assay buffer (0.1 M sodium acetate, 0.1% Triton-X-100, supplemented with ImmunoPure p-nitrophenyl phosphate; Sigma Chemical Co.), and following 3 h of incubation, 10 μL of 1 M NaOH solution was added. The inhibition of the cell growth induced by the tested complexes was detected by measuring the absorbance of each well at 405 nm using a Bio-Rad 680 microplate reader. Mean absorbance for each drug dose was expressed as a percentage of the control untreated well absorbance (T/C) and plotted vs. drug concentration. IC_50_ values, the drug concentrations that reduce the mean absorbance at 405 nm 50% of those in the untreated control wells, were calculated by the four-parameter logistic (4-PL) model. Evaluation was based on means from at least four independent experiments.

#### 3.3.5. Cellular Uptake

HCT-15 cells (2.5 × 10^6^) were seeded in 75 cm^2^ flasks in growth medium (20 mL). After 24 h, the medium was replaced, and the cells were incubated for 24 h with tested complexes. Monolayers were then washed twice with ice-cold phosphate-buffered saline (PBS), harvested, and counted. Cell samples were subjected to five freeze–thaw cycles at −80 °C and then vigorously vortexed. The samples were treated with highly pure nitric acid and transferred into a microwave Teflon vessel. Samples were then submitted to standard mineralization procedures and analyzed for the copper amount by using a Varian AA Duo graphite furnace atomic absorption spectrometer (Varian, Palo Alto, CA, USA) at 324 nm. The calibration curve was obtained using known concentrations of standard solutions purchased from Sigma Chemical Co.

#### 3.3.6. PDI Inhibition

The activity of PDI was assayed by measuring the PDI-catalyzed reduction of insulin in the presence of increasing concentrations of the tested compounds by using PROTEOSTAT PDI assay kit (Enzo Life Sciences, Lausen, Switzerland). Experiments were performed according to the manufacturer’s instructions. Briefly, copper complexes or bacitracin (at increasing concentrations) were added to an insulin PDI solution. Subsequently, DTT was added to start PDI reduction activity, and after 30 min of incubation, the reaction was stopped by adding the stop reagent mixture. The insulin precipitate was labeled with the fluorescent Proteostat PDI detection reagent, and fluorescence intensity was measured at 500 nm excitation and 603 nm emission. IC_50_ values were calculated by the 4-PL model.

#### 3.3.7. Quantification of Thiols

HCT-15 cells (2 × 10^5^) were seeded in a six-well plate in growth medium (4 mL). After 24 h, cells were incubated for 36 h with IC_50_ concentrations of tested compounds. Subsequently, the thiol content was measured as previously described [56].

#### 3.3.8. TEM Analysis

About 10^6^ HCT-15 cells were seeded in 24-well plates, and after 24 h incubation, were treated with IC_50_ concentrations of tested compounds and incubated for an additional 24 h. Cells were then washed with cold PBS, harvested, and directly fixed with 1.5% glutaraldehyde buffer with 0.2 M sodium cacodylate, pH 7.4. After washing with buffer and postfixation with 1% OsO_4_ in 0.2M cacodylate buffer, specimens were dehydrated and embedded in epoxy resin (Epon Araldite). Sagittal serial sections (1 μm) were counterstained with toluidine blue; thin sections (90 nm) were given contrast by staining with uranyl acetate and lead citrate. Micrographs were taken with a Hitachi H-600 electron microscope (Hitachi, Tokyo, Japan) operating at 75 kV. All photos were typeset in Corel Draw 11.

#### 3.3.9. Cell Death Induction

HCT-15 cells were seeded into 8-well tissue-culture slides (BD Falcon, Bedford, MA, USA) at 5 × 10^4^ cells/well (0.8 cm^2^). After 24 h, the cells were washed twice with PBS, and following 24 or 48 h of treatment with IC_50_ doses of the tested compound, cells were stained for 5 min with 10 µg/mL of Hoechst 33258 (20-(4-hydroxyphenyl)-5-(4-methyl-1-piperazinyl)-2,50-bi-1H-benzimidazole trihydrochloride hydrate, Sigma-Aldrich, St. Louis, MI, USA) in PBS. Samples were examined at 5× and 40× magnification in a Zeiss LSM 800 confocal microscope using the Zeiss ZEN 2.3 software system.

#### 3.3.10. Statistical Analysis

All values are the means ± S.D. of no less than three measurements starting from three different cell cultures. Multiple comparisons were made by ANOVA followed by the Tukey–Kramer multiple comparison test (* *p* < 0.05, ** *p* < 0.01), using GraphPad software.

## 4. Conclusions

The methyl ester derivatives **1** and **2** were synthesized and used for the preparation of Cu(I) and Cu(II) complexes **3**–**10**. Concerning the Cu(I) complexes, the lipophilic PPh_3_ and the hydrophilic PTA were selected as coligands to stabilize copper in +1 oxidation state. The compounds were fully characterized both in solid state and in solution. As for the solid-state structural investigation, a multi-technique approach allowed ascertaining the molecular stability of the ligands upon interaction with the copper ions, as well as determining the coordination geometry and copper ions oxidation state. More in detail, X-ray photoelectron spectroscopy data analysis provided information about the electronic and molecular structure of Cu(I) and Cu(II) coordination compounds, evidencing the molecular structure’s stability and confirming the expected oxidation states; X-ray absorption spectroscopy techniques at C, N, and Cu K-edges were applied to deeply investigate the ligand’s functional group stability upon coordination to copper ions (C, N K-edges) as well as the local atomic structure around the copper ion (Cu K-edge). In addition, the complementary information acquired by XAS and XPS allowed disambiguating the monomeric or dimeric chemical structures of Cu(II) complexes **5** and **9**.

All the investigated complexes showed significant cytotoxic effects against a panel of human cancer cell lines, with IC_50_ values in the low/sub-micromolar range and proved to be more effective than the reference metallodrug cisplatin, especially when tested in 3D tumor cell models. Among all derivatives, **4** and **8** which were those most uptook by cancer cells were the most effective in 3D spheroid models, thus attesting that even if no linear correlation was found between the cytotoxic activity and the cellular uptake of all copper compounds, the uptake profile affected efficacy in 3D systems.

Interestingly, mechanistic studies attested their ability to inhibit PDI, an ER protein that is emerging as a very promising cancer-specific molecular target for the development of innovative pharmaceuticals. Coherently, copper complexes induced in cancer cells an outstanding increase in total sulfhydryl content and the induction of ER stress, ultimately leading to paraptotic cancer cell death. Among all, the strongest PDI inhibitors **4** and **9** induced a massive ER cisternae enlargement and a concomitant induction of cytoplasmic vacuolization, which are signs of ER stress and paraptosis induction. In addition, both complexes induced a slight increase in mitochondrial swelling and alteration of cristae features which was found to be not mediated by ROS cellular overproduction.

To the best of our knowledge, this is the first time that PDI is claimed as a putative molecular target for Cu(I) species. Hence, these results open a new perspective scenario for further research validating PDI as a target for Cu(I) complexes and for optimizing copper compound ability to modulate its activity.

## Figures and Tables

**Table 1:** Peak position (eV) and relative assignment of the main features appearing in the C K edge spectra of samples **2**, **5**, and **8** and in the N K edge NEXAFS spectra of samples **2** and **5**.

Sample	2	5	8	Assignment
C K edge			284.0	π*_C=C benzene_
	284.5	284.5	284.5	π*_C=C pyrazole_
	286.1	286.1	286.2	π*_C=N_
	288.3	288.3	288.3	π*_C=O_
	295	295	297	σ*_C-C_
	304	304	303	σ*_C=N_, σ*_C=C_
N K edge	397.9	397.9		π*_1_
	399.5	399.5		π*_2_
	404	403		σ*_C=N_
	411.0	411.0		σ*_C-N_

**Table 2:** Best fit results from the EXAFS analysis of complexes **5** and **9**. The first line reports the multiplicity numbers and average half scattering path lengths as obtained from Avogadro DFT model. In fitting the path, multiplicity numbers were constrained to a molecular model. The lower *R^2^_w_* points out the statistically better fit.

		Cu-N_1_			Cu-Cl			Cu-C_2_ + Cu-N_2_			Cu-C_4_			Cu-N_2_-C_4_ + Cu-C_2_-C_4_			Cu-C_2_-C_4_-N_1_ + Cu-N_2_-C_4_-N_1_			Cu-Cu		
**Sample**	*R^2^_w_* ×10^2^	N	R(Å)	σ^2^(Å^2^) ×10^−3^	N	R(Å)	σ^2^(Å^2^) ×10^−3^	N	R(Å)	σ^2^(Å^2^) ×10^−3^	N	R(Å)	σ^2^(Å^2^) ×10^−3^	N	R(Å)	σ^2^(Å^2^) ×10^−3^	N	R(Å)	σ^2^(Å^2^) ×10^−3^	N	R(Å)	σ^2^(Å^2^) ×10^−3^
**DFT**		2	2.11		2	2.23		4	3.09		4	4.33		12	4.35		8	4.42		1	3.48	
**C5_Mon_**	1.27	2	1.991(5)	5.8	2	2.26(1)	5.6	4	3.00(2)	7.8	4	4.26(2)	2.7	12	4.33(2)	6.3	8	4.68(3)	10.0			
**C9_Mon_**	1.35	2	2.005(5)	7.1	2	2.24(1)	5.2	4	3.03(2)	7.5	4	4.31(2)	2.7	12	4.37(3)	7.3	8	4.68(3)	9.9			
**C5_Dim_**	1.12	2	1.989(5)	5.8	2	2.26(1)	5.5	4	3.00(2)	7.9	4	4.27(2)	3.4	12	4.33(2)	7.6	8	4.69(3)	7.9	0.5(1)	3.53(2)	8.6
**C9_Dim_**	1.58	2	2.072(5)	13.0	2	2.22(1)	8.8	4	3.01(2)	7.5	4	4.29(2)	2.8	12	4.35(2)	6.6	8	4.67(3)	15.0	0.2(1)	3.57(3)	5.5

**Table 3:** Cytotoxicity of ligands **1**–**2**, newly developed copper complexes **3**–**10**, and cisplatin on 2D cell cultures.

	IC_50_ (µM) ± S.D.						
	PSN-1	HCT-15	U1285	A431	2008	C13*	RF
**1**	>50	>50	>50	>50	>50	>50	-
**2**	>50	>50	>50	>50	>50	>50	-
**3**	31.7 ± 4.3	1.7 ± 0.4	11.0 ± 0.3	24.6 ± 2.2	30.9 ± 3.3	5.1 ± 0.3	0.2
**4**	1.6 ± 0.3	6.1 ± 1.6	6.4 ± 1.6	8.4 ± 1.2	3.3 ± 1.4	3.1 ± 0.2	0.9
**5**	1.4 ± 0.2	0.30 ± 0.02	0.70 ± 0.04	1.3 ± 0.4	3.5 ± 1.3	1.2 ± 0.5	0.3
**6**	0.7 ± 0.1	0.4 ± 0.1	0.60 ± 0.01	0.5 ± 0.1	3.2 ± 0.3	0.5 ± 0.2	0.2
**7**	10.6 ± 0.9	0.4 ± 0.1	7.3 ± 1.4	10.5 ± 0.4	4.5 ± 1.2	3.3 ± 0.6	0.7
**8**	11.5 ± 2.1	0.3 ± 0.1	4.7 ± 1.4	10.7 ± 2.1	7.3 ± 1.6	3.7 ± 0.4	0.5
**9**	0.20 ± 0.01	0.20 ± 0.04	0.6 ± 0.1	0.20 ± 0.02	0.4 ± 0.1	0.8 ± 0.01	2.0
**10**	0.5 ± 0.2	0.2 ± 0.1	0.5 ± 0.03	0.4 ± 0.1	1.8 ± 0.2	2.6 ± 0.2	1.4
**cisplatin**	12.1 ± 2.9	15.3 ± 2.6	3.3 ± 1.2	1.7 ± 0.3	2.2 ± 1.0	16.3 ± 3.4	7.4

Cells (3–5 × 10^3^ mL^−1^) were treated for 72 h with tested compounds. Cell viability was estimated by means of the MTT test. The IC_50_ values were calculated by a four-parameter (4-PL) logistic model (*p* < 0.05). S.D. = standard deviation. RF = IC_50_ resistant/IC_50_ parental cells.

**Table 4:** Cytotoxicity of complexes **3**–**10** and cisplatin towards colon HCT-15 cancer cell spheroids.

	IC_50_ (µM) ± S.D.
	**HCT-15**
**3**	28.2 ± 3.0
**4**	10.6 ± 0.4
**5**	25.0 ± 1.1
**6**	27.7 ± 3.3
**7**	42.1 ± 1.6
**8**	5.9 ± 1.3
**9**	40.7 ± 4.0
**10**	13.2 ± 2.8
**cisplatin**	52.6 ± 4.9

Cancer cell spheroids (2.5 × 10^3^ cells/well) were treated for 72 h with tested compounds. Cell viability was estimated by means of the APH test. IC_50_ values were calculated from the dose–response curves by a 4-PL logistic model (*p* < 0.05). S.D. = standard deviation.

## Supplementary Materials

Supplementary materials can be found at https://www.mdpi.com/article/10.3390/ijms23169397/s1.
