# Supplementary material for: Exploring the Antitumor Potential of Copper Complexes Based on Ester Derivatives of Bis(pyrazol-1-yl)acetate Ligands
Source: Int J Mol Sci. 2022 Aug 20;23(16):9397. doi: 10.3390/ijms23169397 (PMC9409343; doi:10.3390/ijms23169397)
Supplement: Supplementary file 1 [file ijms-23-09397-s001.zip › ijms-1865468-supplementary.pdf]

Supplementary material

Exploring the antitumor potential of copper complexes based on ester derivatives of bis(pyrazol-1-yl)acetate ligands

Maura Pellei, Carlo Santini, Luca Bagnarelli, Chiara Battocchio, Giovanna Iucci, Iole Venditti, Carlo Meneghini, Simone Amatori, Paolo Sgarbossa, Cristina Marzano, Michele De Franco, Valentina Gandin

Table of Contents:

Table S1: Detailed SR-XPS data analysis results collected at C1s, N1s, O1s, Cl2p, P2p and Cu2p core levels (Binding Energy (BE), Full Width Half Maximum (FWHM), atomic ratios and assignments), confirming the proposed molecular structures of ligand **2** and complexes **8**, **5** and **9**.

Figure S1: Cu2p spectra of complexes **8** and **9**.

Figure S2: Cellular ROS production.

Figure S3: ^{1}H-NMR spectrum of [HC(pz)$_2$COOCH$_3$] (L^{OMe}, **1**) in CD$_3$CN.

Figure S4: ^{1}H-NMR spectrum of [HC(pz^{Me2})$_2$COOCH$_3$] (L^{2OMe}, **2**) in CD$_3$CN.

Figure S5: ^{1}H-NMR spectrum of [(PTA)Cu(L^{OMe})]PF$_6$ (**3**) in CD$_3$CN.

Figure S6: ^{1}H NMR spectrum of [(PPh$_3$)Cu(L^{OMe})]PF$_6$ (**4**) in CD$_3$CN.

Figure S7: ^{1}H-NMR spectrum of [(PTA)Cu(L^{2OMe})]PF$_6$ (**7**) in CD$_3$CN.

Figure S8: ^{1}H NMR spectrum of [(PPh$_3$)Cu(L^{2OMe})]PF$_6$ (**8**) in CD$_3$CN.

Table S1. Detailed SR-XPS data analysis results collected at C1s, N1s, O1s, Cl2p, P2p and Cu2p core levels (Binding Energy (BE), Full Width Half Maximum (FWHM), atomic ratios and assignments), confirming the proposed molecular structures of ligand **2** and complexes **8**, **5** and **9**.

Table S1. XPS data analysis results: BE, FWHM values and Assignments for samples **2**, **8**, **5** and **9**.

Sample	Signal	BE (eV)	FWHM (eV)	atomic ratios	Assignment
**2**	C1s	284.70	1.44	14.1	C-C aromatic + aliphatic
		286.25	1.44	2.2	C-N
		287.79	1.44	1.1	C-O-C
		289.13	1.44	1.0	COOR
	N1s	399.88	2.14	1.1	C-N
		401.77	2.14	1.0	C=N
	O1s	532.07	1.59	1.8	C=O (+ impurities)
		533.25	1.59	1.0	C-O
**8**	C1s	284.70	1.80	9.5	C-C aromatic + aliphatic
		285.84	1.80	3.4	C-N
		287.48	1.80	1.0	C-O-C
		288.78	1.80	1.0	COOR
	N1s	399.85	2.80	1.0	C-N
	O1s	531.01	2.69	1.2	C=O (+ impurities)
		533.11	2.69	1.0	C-O
	$P2p_{3/2}$	131.89	3.31	1.0	$R-PPh_3$
	$Cu2p_{3/2}$	932.20	3.31	1.0	Cu(I) in coordination compound
**5**	C1s	284.70	1.45	6.8	C-C aromatic + aliphatic
		286.05	1.45	2.0	C-N
		287.41	1.45	1.0	C-O-C
		288.84	1.45	1.0	COOR
	N1s	400.92	3.20	1.0	C-N
	O1s	532.05	2.08	2.1	C=O (+ impurities)
		533.43	2.08	1.0	C-O
	$Cl2p_{3/2}$	199.27	2.61	0.8	Cl-Cu monomeric coord. compound
		201.31	2.61	1.0	Cu-Cl-Cu dimeric coord. compound
	$Cu2p_{3/2}$	932.19	2.53	1.0	Cu(I)?
		934.82	2.53	0.8	Cu(II) in coordination compound
**9**	C1s	284.70	1.52	14.3	C-C aromatic + aliphatic
		286.23	1.52	2.6	C-N
		287.71	1.52	1.0	C-O-C
		289.12	1.52	1.0	COOR
	N1s	400.34	2.85	1.0	C-N
	O1s	532.15	1.67	3.8	C=O (+ impurities)
		533.44	1.67	1.0	C-O
	$Cl2p_{3/2}$	199.11	1.86	1.0	Cl-Cu monomeric coord. compound
	$Cu2p_{3/2}$	932.24	2.54	1.0	Cu(I)?
		935.24	2.54	2.2	Cu(II) in coordination compound

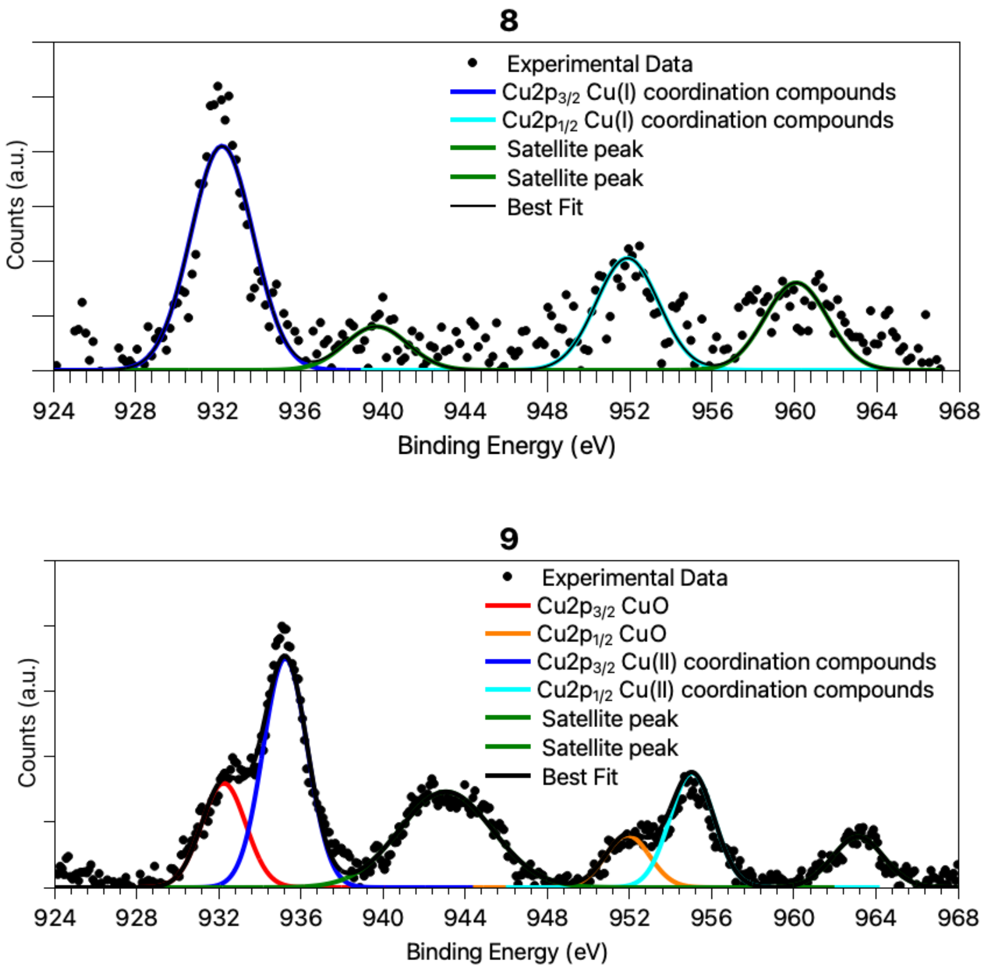

Figure S1. Cu2p spectra of complexes **8** and **9**.

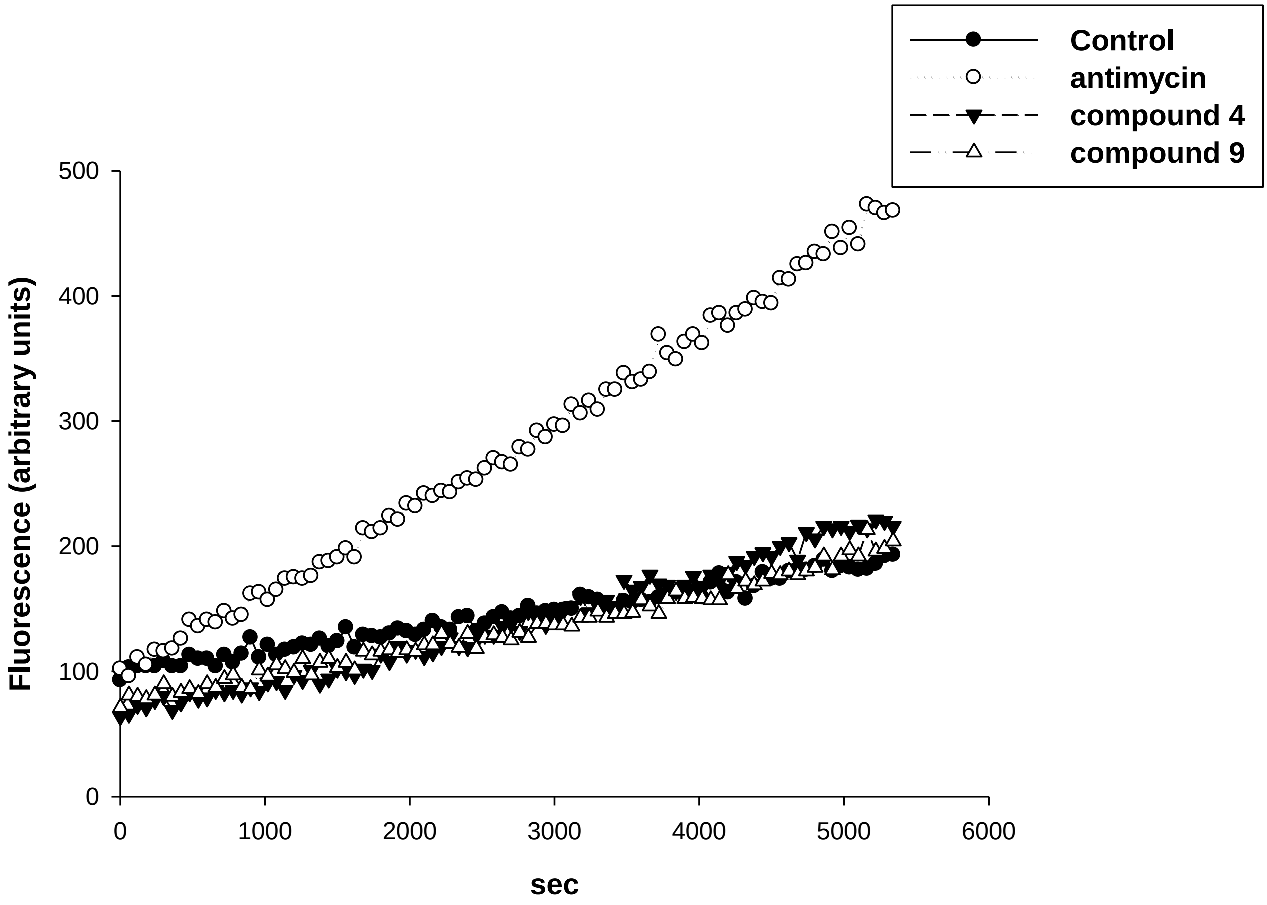

Figure S2. Cellular ROS production. Effect of tested compounds or antimycin on hydrogen peroxide formation in HCT-15 colon cancer cells. Error bars indicate S.D.

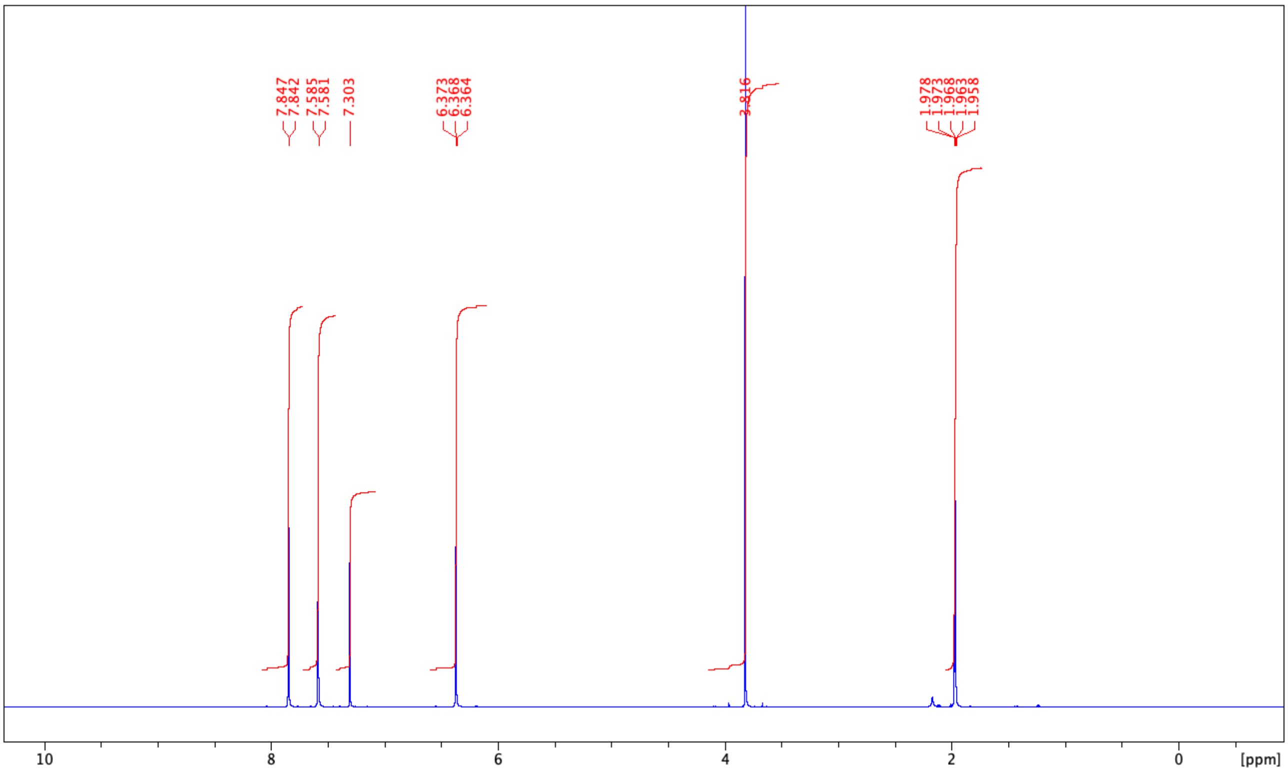

Figure S3: ^1H-NMR spectrum of [HC(pz)$_2$COOCH$_3$] (L^{OMe}, **1**) in CD_3CN.

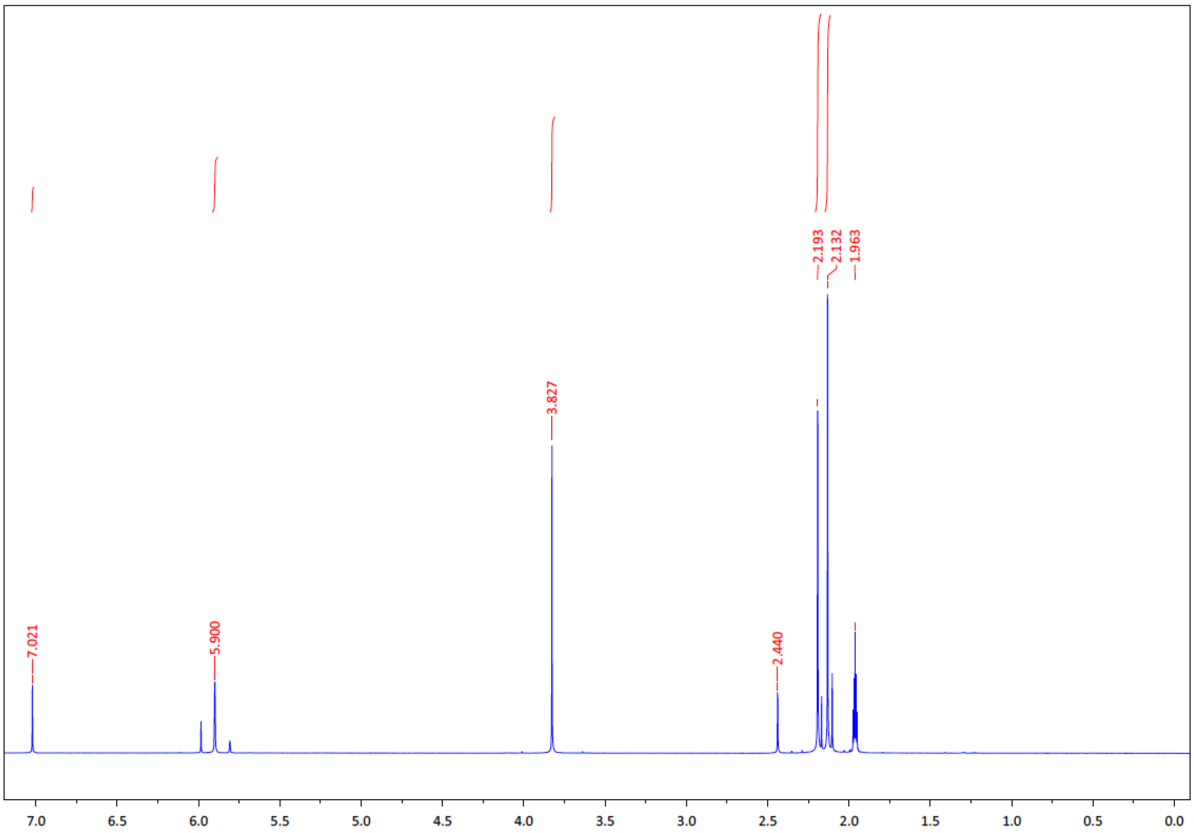

Figure S4: ^1H-NMR spectrum of [HC(pz^{Me2})$_2$COOCH$_3$] (L^{2OMe}, **2**) in CD_3CN.

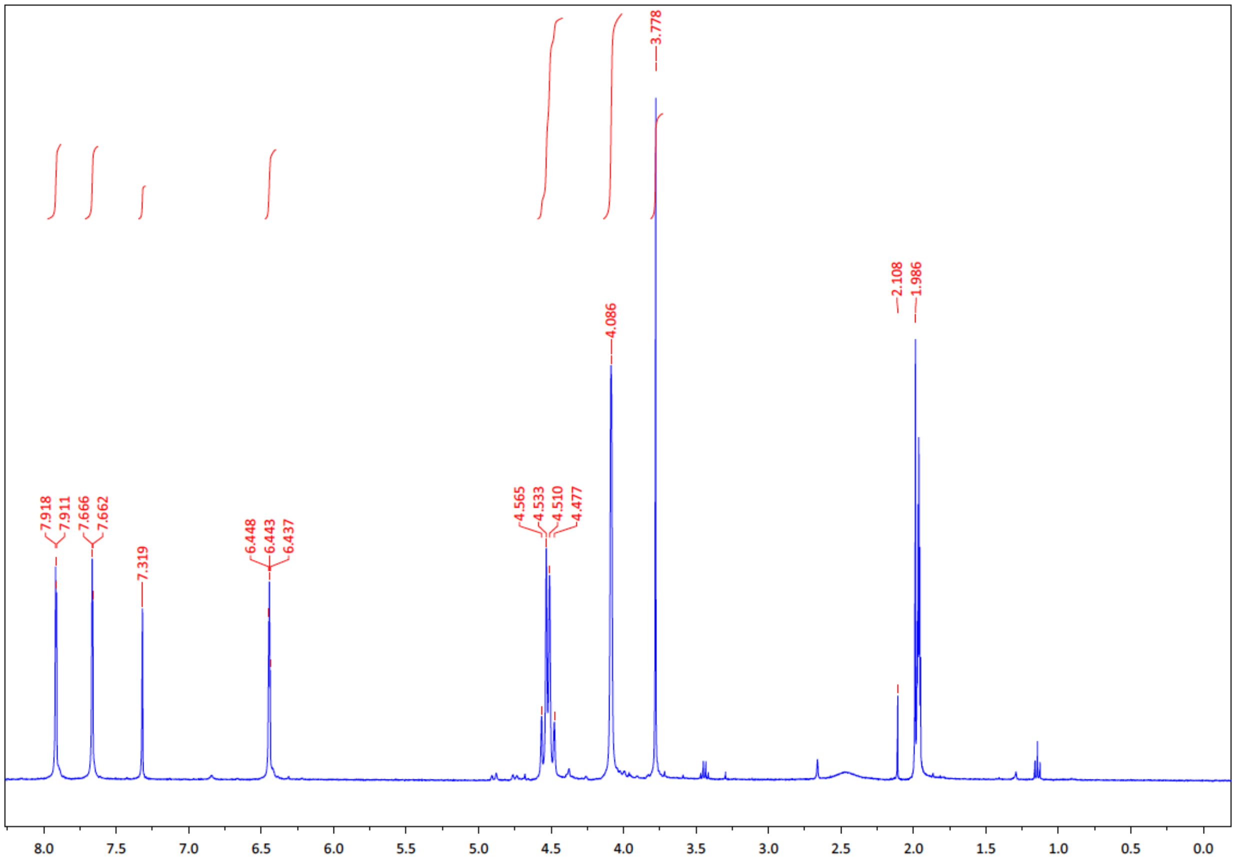

Figure S5: ^{1}H-NMR spectrum of [(PTA)Cu(L^{OMe})]PF_6 (**3**) in CD_3CN.

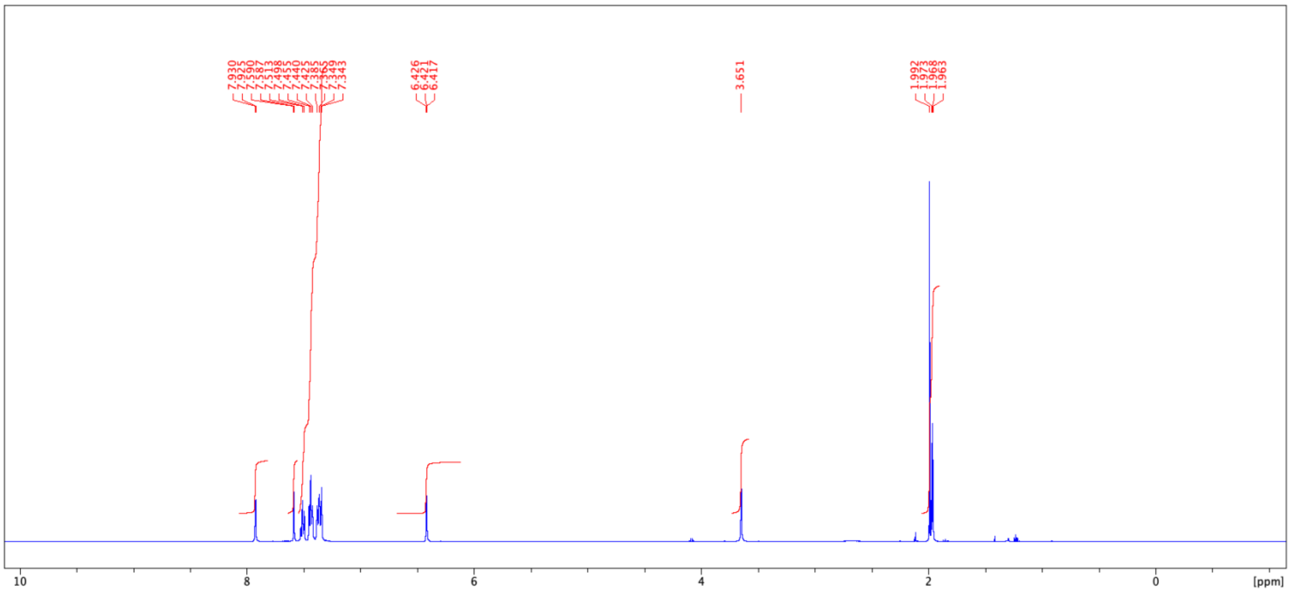

Figure S6: ^{1}H-NMR spectrum of [(PPh_3)Cu(L^{OMe})]PF_6 (**4**) in CD_3CN.

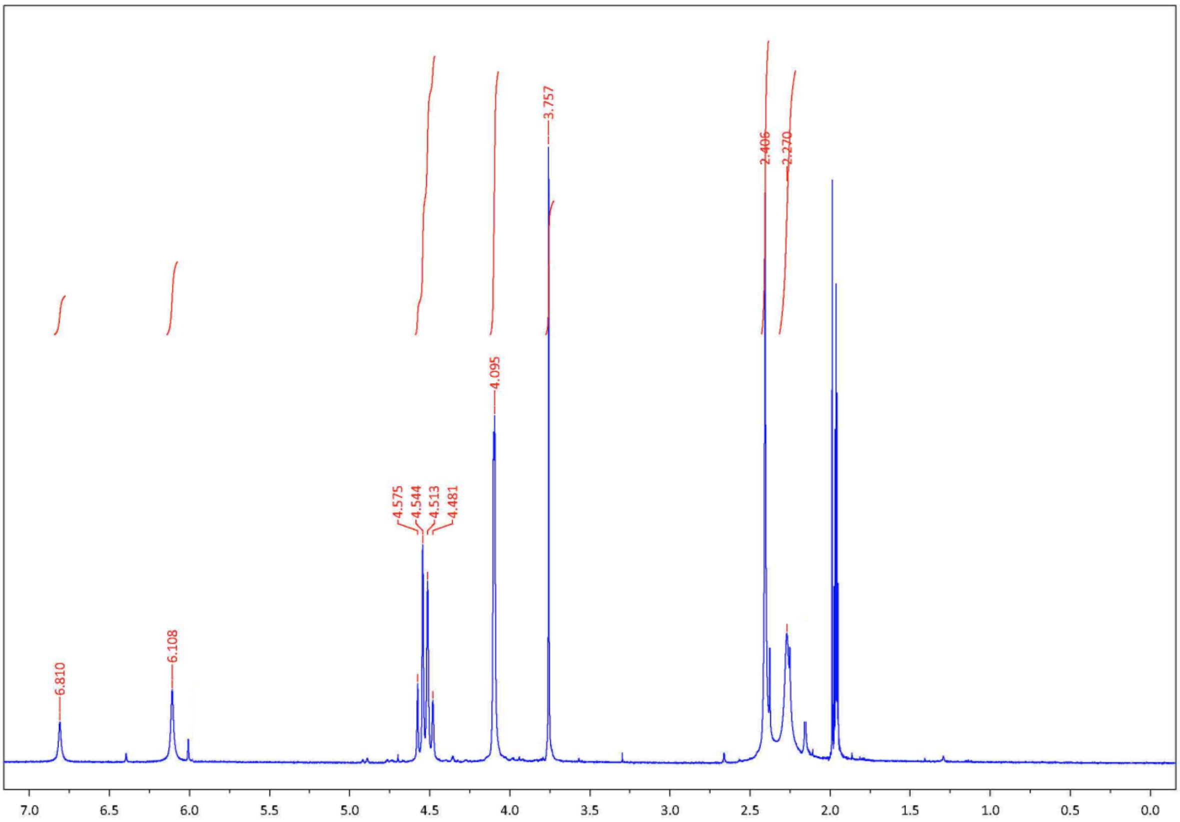

Figure S7: ^{1}H-NMR spectrum of [(PTA)Cu(L^{2OMe})]PF_6 (**7**) in CD_3CN.

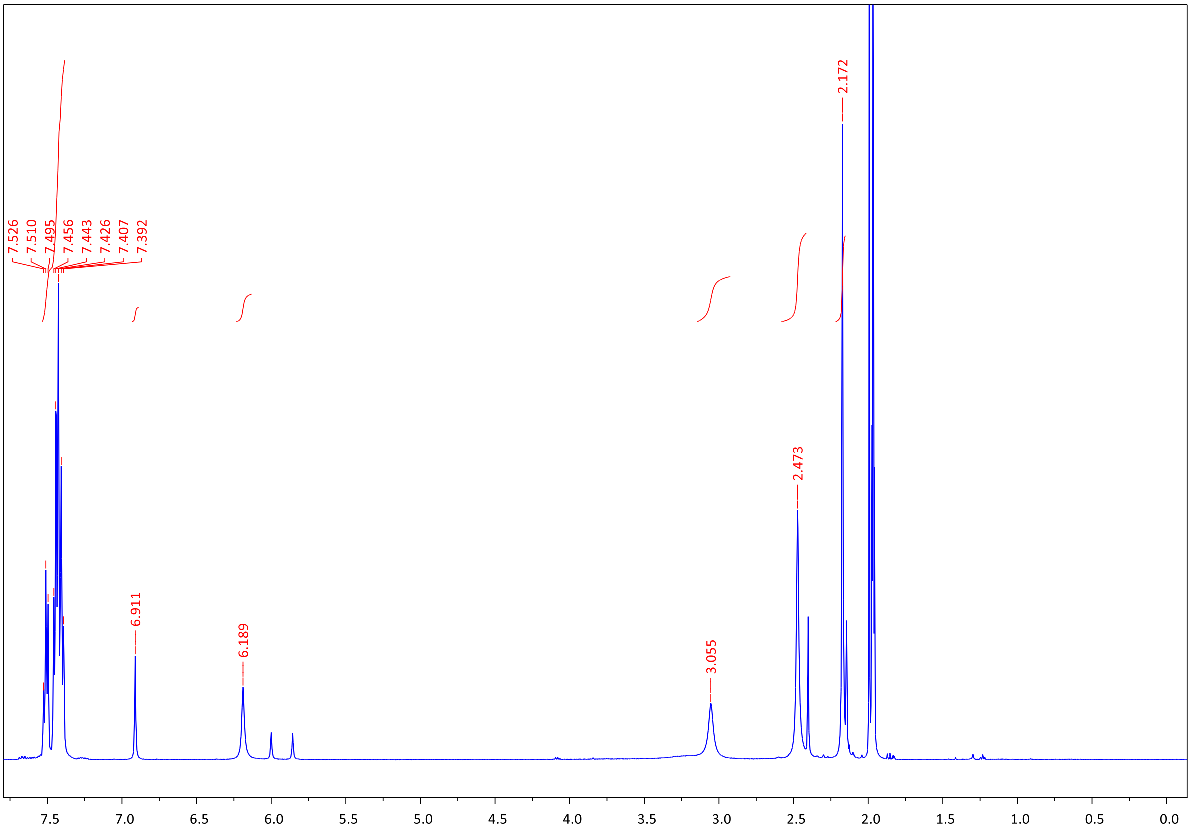

Figure S8: ^{1}H NMR spectrum of [(PPh_3)Cu(L^{2OMe})]PF_6 (**8**) in CD_3CN.
